# Immune checkpoint modulators in cancer immunotherapy: recent advances and emerging concepts

**DOI:** 10.1186/s13045-022-01325-0

**Published:** 2022-08-17

**Authors:** Yuchen Wang, Hao Zhang, Chao Liu, Zeyu Wang, Wantao Wu, Nan Zhang, Longbo Zhang, Jason Hu, Peng Luo, Jian Zhang, Zaoqu Liu, Yun Peng, Zhixiong Liu, Lanhua Tang, Quan Cheng

**Affiliations:** 1 Department of Neurosurgery, Xiangya Hospital, Center South University, Changsha, 410008 Hunan People’s Republic of China; 2 Xiangya School of Medicine, Central South University, Changsha, People’s Republic of China; 3 Department of Oncology, Xiangya Hospital, Central South University, Changsha, 410008 Hunan People’s Republic of China; 4 One-Third Lab, College of Bioinformatics Science and Technology, Harbin Medical University, Harbin, People’s Republic of China; 5 Department of Neurosurgery, and Department of Cellular & Molecular Physiology, Yale University School of Medicine, New Haven, USA; 6 Department of Neonatology, Yale University School of Medicine, New Haven, USA; 7 Department of Oncology, Zhujiang Hospital, Southern Medical University, Guangzhou, People’s Republic of China; 8 Department of Interventional Radiology, The First Affiliated Hospital of Zhengzhou, Zhengzhou, People’s Republic of China; 9 Department of Geriatrics, Xiangya Hospital, Central South University, Changsha, People’s Republic of China; 10 Department of Neurosurgery, The Second Affiliated Hospital, Chongqing Medical University, Chongqing, People’s Republic of China; 11 National Clinical Research Center for Geriatric Disorders, Xiangya Hospital, Central South University, Changsha, People’s Republic of China; 12 Department of Neurosurgery, Central Hospital of Zhuzhou, Zhuzhou, People’s Republic of China

**Keywords:** Immunotherapy, Immune checkpoint, Clinical trial, Targeted drugs

## Abstract

The discovery of immune checkpoint inhibitors (ICIs) has now been universally acknowledged as a significant breakthrough in tumor therapy after the targeted treatment of checkpoint molecules: anti-programmed cell death protein 1/programmed cell death ligand 1 (PD-1/PD-L1) and anti-cytotoxic T lymphocyte-associated antigen-4 (CTLA-4) on several cancer types achieved satisfying results. However, there are still quite a lot of patients suffering from severe side effects and ineffective treatment outcomes. Although the current ICI therapy is far from satisfying, a series of novel immune checkpoint molecules with remarkable preclinical and clinical benefits are being widely investigated, like the V-domain Ig suppressor of T cell activation (VISTA), which can also be called PD-1 homolog (PD-1H), and ectonucleotidases: CD39, CD73, and CD38, which belong to the ribosyl cyclase family, etc. In this review, we systematically summarized and discussed these molecules' biological structures, molecular features, and the corresponding targeted drugs, aiming to help the in-depth understanding of immune checkpoint molecules and promote the clinical practice of ICI therapy.

## Background

Immunotherapy has shown great potential and power in cancer treatment over the past decades. Multiple studies have demonstrated its efficacy in inhibiting the progression of malignancies. The prosperity of chimeric antigen receptor T‐cells (CAR-T) therapy against multiple cancers revolutionized cancer immunotherapy in the year 2013 [1]. Immune checkpoint inhibitors (ICIs) [2], known for Drs. James Allison’s and Tasuku Honjo’s unprecedented discovery of CTLA-4 and PD-1 [3], have been widely investigated and applied in clinical practice.

Two specific signals are vital for T cells’ full function: the interaction between the antigenic peptide/major histocompatibility complex (MHC) on the surface of antigen-presenting cells (APCs) with the T cell receptor (TCR), and the antigen-independent co-signaling molecules. The latter are the so-called immune checkpoints [4]. Ordinarily, checkpoints like CD28 belong to co-stimulators. When binding to its ligand, CD28 stimulates T cells to proliferate and be recruited to the specific region. On the contrary, co-inhibitors such as PD-1 play the opposite role [5]. Co-inhibitors and co-stimulators orchestrate the cell-mediated immune responses in the human body. Besides, most antigens expressed on tumor cells are not only neo-antigens presented explicitly on cancer cells but also self-antigens (tumor-associated antigens and cancer–testis antigens) simultaneously expressed on cancer and normal cells [6]. Cancer–testis antigens could be categorized into chromosome X-mapped antigens, including MAGE-A, BAGE, NY-ESO-1, and IL-13Rα, which have been broadly studied, and non-chromosome X-mapped antigens [7]. It should be noted that high-affinity TCRs for self-antigens are preferentially depleted because of positive selection, and the affinities of the remaining TCRs for self-antigens are lower than those for neo-antigens [8]. Therefore, the low immune responses toward tumor cells, the so-called immune escape, could be described as self-protection since TCRs were generally reported to interact with neo-antigens instead of self-antigens [9]. Advanced studies showed the hypoxia and ischemia condition in the tumor microenvironment (TME) triggered anti-inflammatory molecules rocketing [4], which indicated the suppression of co-stimulators and the hyperfunction of co-inhibitors in TME could potentially mediate immune escape. Given the vital role of immune checkpoints in regulating immune response, a series of immune checkpoint inhibitors are developed [10, 11]. The core part of the therapeutic effect lies in re-activating the patients’ immune system to enhance primary anti-tumor activity.

This review will first summarize the most widely studied immune checkpoints: CTLA-4, PD-1, and PD-L1. Then, we will focus on novel immune checkpoints that have been explored, including V-domain Ig suppressor of T cell activation (VISTA), T cell immunoglobulin and mucin domain-containing protein 3 (TIM-3), lymphocyte activation gene 3 (LAG-3), indoleamine 2,3-dioxygenase 1 (IDO-1), CD161, CD73, CD38, CD39, CD93, CD47, BTLA, CD70, VTCN1, and B7-H3. In brief, CD73 could cooperate with CD39 or CD38 to downregulate the level of ATP and upregulate adenosine, while CD38 also interposes the NAD+ signaling pathway. IDO-1 mediates the transformation from Trp to Kyn. VISTA restrains cytokine secretion. TIM-3 facilitates intracellular calcium influx. All these molecules could lead to T cell exhaustion. CD47 and BTLA both have ITIM and ITSM domains, but CD47 inhibits phagocytosis through dephosphorylating motor protein myosin. BTLA then blocks TCR from working. LAG-3 also hinders CD4+ T cells activation by tying to MHC-II against CD4. Once CD161 is banded to LLT1, the complex would inhibit NK cell activation. In contrast, the CD27-CD70 combination plays the opposite role by increasing IFN-γ expression and igniting the Akt signaling pathway on NK cells. CD93 stresses tumor angiogenesis. B7-H3 and B7-H4 need more fundamental studies. These newly characterized immune checkpoints and their ongoing or completed clinical studies will be systematically summarized, which could help suggest a promising future for clinical application.

## Classical immune checkpoints

CTLA-4 (CD152) is a classical immune checkpoint molecule [12], which is closely associated with CD28 but plays different roles in the immune response. Locating on CD4+ as well as CD8+ T cell surfaces, CD28 is a costimulatory receptor. When interacting with the ligands (B7): CD80 dimer and CD86 monomer, a signal will be sent, along with the signal from TCR, to activate the whole cell. CTLA-4 was predominantly found in intracellular vesicles and compared with CD28. It has a higher affinity with CD80 and CD86, competing with CD28 for binding ligands [13]. Subsequently, the CTLA-4-CD80 complex or CTLA-4-CD86 complex will be transported to the cytoplasm and eliminated by lysosomal compartments, which can eventually suppress the T cell activation [12]. Ipilimumab has been the optimal anti-CTLA-4 antibody despite the potential hyperfunction of the immune system. It is a fully human antibody targeting CTLA-4 and has received FDA approval as the first available ICI for treating patients with metastatic melanoma in 2010 [14]. With a molecular mass of approximately 148 kDa, Ipilimumab is composed of four polypeptide chains—two identical heavy chains of 447 amino acids and two identical kappa light chains of 215 amino acids [15]. It is generally thought that Ipilimumab works by blocking the interaction between B7 and CTLA-4 and then functioning in lymphoid organs. However, the novel study showed that under physiologically relevant conditions, the blockage of the B7-CTLA-4 complex could be rarely found. Ipilimumab achieved its effect in the experiment without breaking the B7-CTLA-4 interaction [16].

Human PD-1, also called CD279, is encoded by the PDCD1 gene and belongs to the immunoglobulin gene superfamily [17]. PD-1 is a type I transmembrane glycoprotein containing a single extracellular IgV domain, a hydrophobic transmembrane domain, and a cytoplasmic tail structure domain, mainly found on activated T cells [1, 18]. After incorporating with its ligands, PD-L1, and PD-L2, which usually overexpress on cancer cells, the whole complex will turn into a “brake,” downregulating the activity of signaling pathways like PI3K/AKT or Ras/MEK/ERK, thus impairing T cell proliferation as well as activation. Besides, PD-L1 is also expressed on tumor-infiltrating lymphocytes (TILs) like helper T (Th) cells or regulatory T cells (Tregs), leading to a T cell exhaustion caused by PD-L1 overexpression on cancer cells and TILs [19]. TILs refer to a series of cells, including T cells, B cells, macrophages, and natural killer (NK) cells that shape the TME and affect tumor proliferation. High-intensity TILs typically imply higher anti-tumor activities and a better prognosis. Notably, this signal transduction could be used by the human body to restrict the range and level of inflammatory response and by tumor cells to escape immune response [20]. Since Pembrolizumab, as a humanized monoclonal anti-PD-1 antibody, was approved by FDA for treating patients with advanced melanoma and non-small-cell lung cancer (NSCLC) in 2014 [21], at least six more anti-PD-1 or anti-PD-L1 antibodies have been approved then put into clinical application [17]. However, along with the wide usage of antibodies, taking Pembrolizumab as an example, many side effects have been reported, including hypophysis [22], hypothyroidism [23], rash, fatigue, pneumonitis, hepatotoxicity, colitis [24], and type I diabetes [25]. General ICIs have already been approved safe with a toxicity profile favorable to conventional chemotherapy. The acquired resistance to the antibodies following ICI therapy has also been reported [26]. Overall, overexpression of PD-L1 is still recognized as a critical suppression of anti-cancer immunity, though regulators targeting PD-L1 have not reached the desired effect. Further studies should be conducted on more powerful PD-L1 regulators to aim for efficacy-strengthened immunotherapy [27].

As for the two classical immune checkpoints, some studies suggested that conjoint immunotherapy, including anti-PD-1 and anti-CTLA-4, would exhibit superior anti-tumor responses compared with single-agent therapy [13]. The theoretical basis that lies in each checkpoint has its unique pathway that can work independently [28], though nowadays, studies show interesting cross talk between them, which indicates an excess effect [13]. Until now, related studies have been conducted in a phase I trial in which Nivolumab combined with Ipilimumab was administered to advanced melanoma patients. The result showed a 40% objective response rate for 53 patients who received concurrent Nivolumab/Ipilimumab and reversible 3–4 related adverse events similar to what has been reported in historical monotherapy experience [29], revealing concurrent Nivolumab/Ipilimumab had a manageable safety profile and achieved promising clinical effect. In another study in melanoma (NCT01844505), investigators found the median progression-free survival (PFS) was 11.5 months for Nivolumab plus Ipilimumab rather than 2.9 months for Ipilimumab alone and 6.9 months for Nivolumab alone. The difference continued to objective response rates assessment, where the rates were 43.7%, 19.0%, and 57.6% in the patients who received Nivolumab or Ipilimumab only, otherwise being conducted with Nivolumab plus Ipilimumab, respectively. Besides, combinations of Nivolumab and Ipilimumab have demonstrated promising clinical benefits in NSCLC (NCT02477826) [30], pleural mesothelioma (NCT02899299) [31], liver cancer (NCT03222076) [32], colorectal cancer (CRC, NCT03350126) [33], and renal cell carcinoma [34] (RRC) (NCT02231749). To summarize, combining Nivolumab and Ipilimumab is a potential treatment option for previously untreated advanced melanoma, NSCLC, and RRC [35].

Previous studies have also demonstrated that radiotherapy could cooperate reasonably with anti-PD-1/anti-PD-L1 therapies. Firstly, radiotherapy promoted tumor-infiltrating lymphocytes and expanded the TCR repertoire in the TME [36, 37]. Secondly, radiotherapy upregulated PD-L1 expression on tumor cells, which provided targets for anti-PD-1/anti-PD-L1 therapy [38]. Thirdly, radiotherapy increased MHC-I expression on tumor cells and relieved resistance to anti-PD-1/anti-PD-L1 therapy [39]. Radiotherapy was also reported to induce a better response to lung cancer against anti-CTLA-4 therapy [40]. Besides, chemotherapy has been widely explored as an appropriate partner with anti-PD-1/anti-PD-L1 and anti-CTLA-4 therapies based on the immune modulatory effect of chemotherapeutic agents. For instance, carboplatin and pemetrexed with Pembrolizumab significantly improved PFS for advanced non-squamous NSCLC in a randomized phase II study [41]. Also, it has been reported that Keynote189, a phase III study concerning combination therapy of pemetrexed, platinum chemotherapy, and Pembrolizumab (MK-3475) on participants with first-line metastatic NSCLC, owned its role as first‐line standard‐of‐care therapy with metastatic non-squamous NSCLC [42]. Another study (Checkmate9LA, NCT03215706) confirmed that combination therapy of Nivolumab plus Ipilimumab, along with two cycles of chemotherapy, could significantly improve patients' outcomes in NSCLC, despite PD-L1 levels. It also improved the survival rates of patients from the experimental group in the early stage of the study compared to the study of checkmate227 [43]. In addition, local Melphalan combined with Ipilimumab resulted in a durable response in advanced melanoma patients [44]. These remarkable results demonstrated the great potential of anti-PD-1/anti-PD-L1 and anti-CTLA-4 therapies in clinical management.

## Novel immune checkpoints

In addition to PD-1 and CTLA-4, a wealth of new immune checkpoint targets have emerged continuously (Table 1). VISTA has been found to express on resting CD4+ T cells to act as a coinhibitory receptor and could negatively regulate T cell activation [45–48]. CD161 was encoded by KLRB1 and mainly expressed on CD8+ T cells. The CD161 activation was triggered by CLEC2D (C-type lectin domain family 2 member D) and suppressed the anti-cancer capacity of T cells [49]. TIM-3, mainly expressed by interferon-γ (IFN-γ)-producing CD4+ and CD8+ T cells, could bind with its ligands, galectin-9, phosphatidylserine (PtdSer), and CEACAM-1, triggering phosphorylation of Tyr256 and Tyr263 by the tyrosine kinase ITK [50]. LAG-3, mainly expressed by activated T and NK cells, could bind with its ligands, major histocompatibility complex class II (MHC-II), and fibrinogen-like protein 1 (FGL1), inhibiting the interaction between LAG-3 and MHC-II [51]. CD39, also called ecto-nucleoside triphosphate diphosphohydrolase-1 (ENTPD-1), together with CD73/ecto-5′-nucleotidase and CD38, is a multifunctional cell protein mainly expressed on immune cells, catalyzing the conversion of nicotinamide adenine dinucleotide (NAD+) to adenosine diphosphate ribose (ADPR) when working as an enzyme [52–54] and regulating the extracellular adenosine when working as a vital intercellular signaling molecule [55]. B7-H3 is a type I transmembrane protein and found aberrantly expressed in a high proportion of human malignancies. It reduces type I IFN released by T cells and downregulates the cytotoxic activity of NK cells [56, 57]. Cell surface lectin CD93, predominantly expressed on endothelial cells, selectively marks and essentially maintains LSCs (leukemia stem cells) through silencing of CDKN2B (cyclin-dependent kinase inhibitor 2B), a significant cell cycle inhibitor, which makes CD93 a primary target to acute myeloid leukemia (AML) [58].

**Table 1:** Comparison of properties of novel immune checkpoint modulators

Target	Chromosomal location	Binding partner	Expression
VISTA	10q22.1	VSIG3	Myeloid cells, T cells
CD38	4p15.32	CD31	Non-hematopoietic cells, immune cells
CD39	10q24.1	Unknown	B cells, NK cells, DCs, monocytes, macrophages, Tregs
CD73	6q14.3	Unknown	endothelial cells, lymphocytes, tumor cells, stromal cells
LAG-3	12p13.31	MHC-II, galectin-3, LSECtin, a-synuclein, FGL1	Activated T cells, B cells, Tregs, NK cells, DCs
IDO-1	8p11.21	AhR	DCs, eosinophils, tumor cells
CD27	12p13.31	CD70	T cells, B cells, plasma cells, NK cells
TIM-3	5q33.2	Galectin-9, Ceacam-1, HMGB1, PtdSer	Activated T cells, B cells, Tregs, DCs, NK cells, monocytes
CD47	3q13.12	SIRPA	Human cells, tumor cells
CD93	20p11.21	IL-17D	Endothelial cells
CD161	12p13.31	LLT1	NK cells, T cells
BTLA	3q13.2	HVEM	Mature B cells, T cells, Tregs, macrophages, DCs
VTCN1	1p13.1-p12	Unknow	Antigen-presenting cells
B7-H3	15q24.1	Unknow	Activated T cells, NK cells, DCs, monocytes, tumor cells
TIGIT	3q13.31	CD155, CD112, CD113	NK cells, activated T cells, Tregs, follicular T helper cells

## VISTA

### Structure and function

**Fig. 1:**
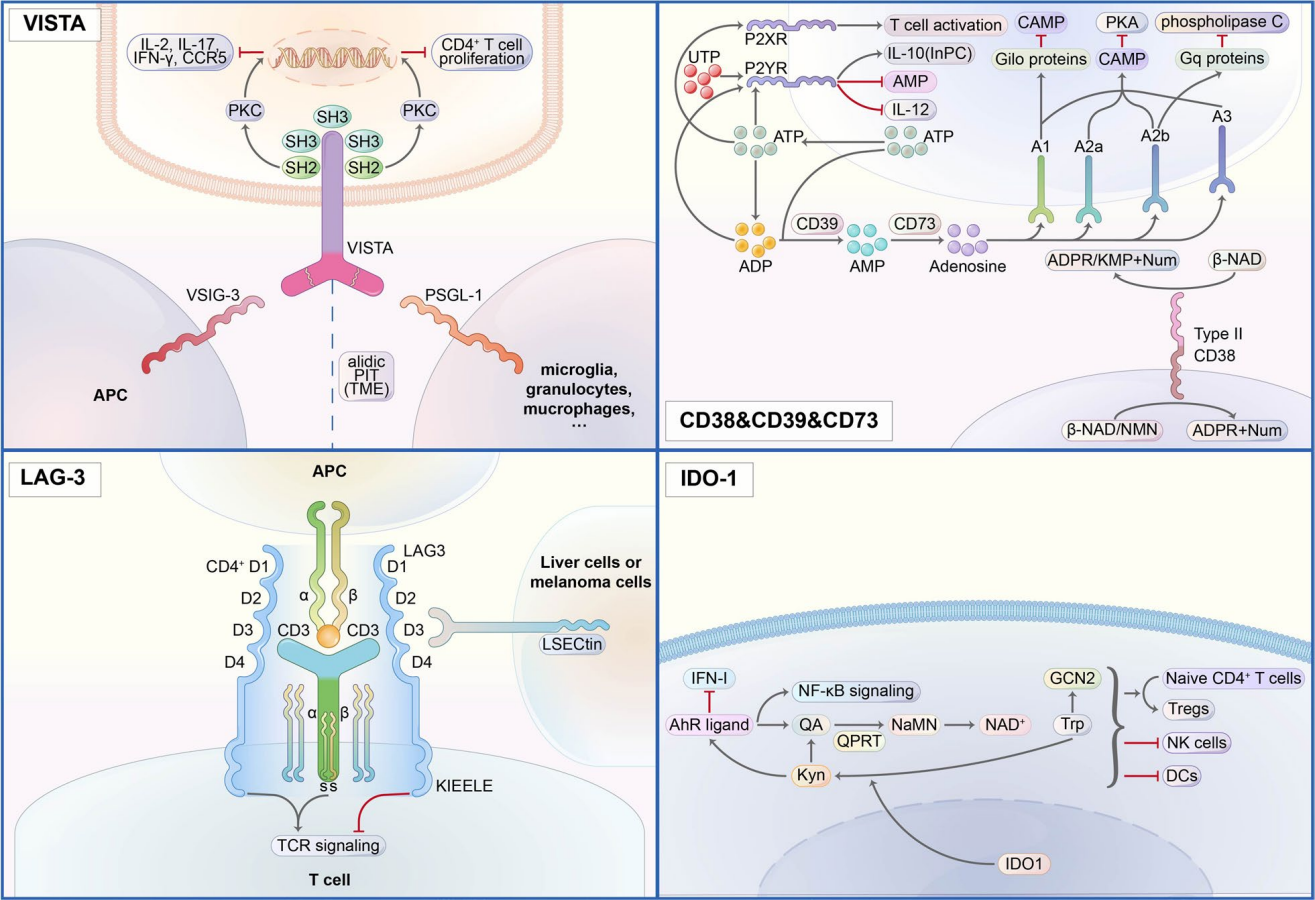
Mode of action of VISTA, CD38/CD39/CD73, LAG-3, and IDO-1 signaling pathways

VISTA is a type I transmembrane protein with 279 amino acids (AAs). It contains an extracellular domain with 162 AAs, a transmembrane domain with 21 AAs, and a cytoplasmic domain containing 96 AAs [59] (Fig. 1). The extracellular region could be divided into two parts, one is an immunoglobulin (Ig) V domain with a single N-terminal, and the other is about 30 AAs stalk. An analysis showed that among the B7 family members, VISTA had been proved to have its highest homology with PD-L1, and the sequence identity could reach 22% [60]. Genetically, it is chromosome 10(10q22.1) where we found VISTS expression, and no neighboring Ig superfamily members could be found. All of this may partly explain the relatively low similarity. Among B7 members, VISTA is the most conservative, besides 76% identity was observed when considering mice and humans, and it is more likely that the translocation springs up along with evolution [45, 61]. Between the assumed B and F strands, the canonical disulfide bond could be found by the IgV domain of VISTA. Besides, VISTA has four additional unique invariant cysteines. VISTA shows its homology with CD28 and CTLA-4. Although lacking a classic ITIM/ITAM motif inside the conserved cytoplasmic tail, VISTA still plays a ligand and a receptor in regulating immune responses. The function was achieved by three C-terminal Src homology domain 3 (SH3) binding motifs, an Src homology domain 2 (SH2) binding motif detected in the medial cytoplasmic tail, multiple casein kinase 2, and phosphokinase C phosphorylation that was in the cytoplasmic domain [60, 62]. The hematopoietic compartment, especially the myeloid cells, is where VISTA mostly be found. To be detailed, VISTA can be found in microglia, monocytes, neutrophils, macrophages, and dendritic cells (DCs). PSGL-1 and VSIG3 have been identified as VISTA’s confirmed ligands. VSIG8-VISTA binding is relatively weak. It is a physiological condition where VISTA can interact with VSIG3, and PSGL-1 is bound to VISTA on T cells in an acidic environment [63]. The VSIG3-VISTA complex will downregulate the level of multiple cytokines, such as interleukin (IL)-2, IL-17, interferon γ (IFN)-γ, and chemokine (C–C motif) ligand 5 (CCL5). Glycosylation and tyrosine sulfation regulate the function of the compound consisting of PSGL-1, selectins, and VISTA, while the modification of the compound is measured by lymphocyte activation. PSGL-1 is highly linked with T cell exhaustion [59, 60, 62].

### Clinical trials on VISTA (Table 2)

Since multiple preclinical models have proved the high therapeutic effectiveness of VISTA in restricting tumor proliferation, VISTA-targeted antagonists have stepped forward into the clinical trial [64, 65].

- CA-170 is one of the first agents for a clinical trial. As PD‐L1, PD‐L2, and VISTA antagonist, CA-170 is uncovered to work with silenced T cells and block cytokine secretion by recognizing the binding sites conserved in VISTA [60, 64]. It is the first oral immune checkpoint inhibitor, and data from the clinical trial (NCT02812875) ensured its pharmacological safety and effectiveness. The completed phase I study was conducted on 71 adult patients suffering from advanced solid tumors or lymphomas who progressed or were non-responsive to available therapies. Participants were given CA-170 orally once or twice daily. The result showed an acceptable safety profile and a relatively shorter pharmacokinetics (PK) exposure with a t1/2 of 3.4 h for CA-170. Evidence of peripheral T cell activation was proved by the increased proportion of circulating CD8+ and CD4+ T cells expressing activation markers, CD69 and Granzyme B and OX-40 (CD134).
- Another monoclonal antibody that blocks the immune inhibition of VISTA, VSTB112, is also under commercial and therapeutic development [60]. As an anti‐VISTA monoclonal antibody (mAb), VSTB112 links to an epitope consisting of C–C′ loops and adjacent helix, VISTA with VSIG3 and PSGL-1 [63]. The phase I trial on the anti-VISTA antagonist, VSTB112 (JNJ-61610588, NCT02671955), was launched in January 2016 by Janssen Research & Development. In this trial, participants were given intravenous infusions of JNJ-61610588 until disease progression. Unfortunately, the whole research was terminated due to financial concerns.
- SG7 is a species cross-reactive antibody against murine, cynomolgus monkey, and human VISTA with high affinity [63]; the binding between VISTA and SG7 relies on H122 and E125, located on the histidine-rich tip of VISTA, blocking the bination of VISTA and PSGL-1/VSIG3. In the study, researchers chose splenocytes from C57BL/6 mice that were activated by anti-CD3/anti-CD28 beads and incubated them with mVISTA-Fc in a complex with SG7 concentration gradient at pH 6.0, then found the binding of mVISTA-Fc with mouse T cells was blocked by SG7 in a dose-dependent manner, where SG7 completely stopped the interaction at the dose of > 10 nM. In human T cells, a dose-dependent reaction was observed when activated CD4+/CD8+ cells were incubated to hVISTA-Fc, in which > 50 nM SG7 also suppressed the hVISTA/T cell bination [59]. Till now, at least 24 clinical trials have been registered on ClinicalTrials.gov, though no result has been reported.

## CD73, CD39, and CD38

### Structure and function

Intercellular signaling relies on specific molecules, among which adenosine has been identified as one of the regulators of multiple physiological and pathological processes [66]. Adenosine is a nucleoside derived from the extracellular hydrolysis of adenine nucleotides. Since adenine nucleotides play a core role in the biosynthesis of ATP, rapidly developed adenosine would indicate ischemia and anoxia, which is most likely to be found in the inflammation region. Adenosine was primarily thought to be a potent vasodilator in 1927 [66]. At the same time, advanced studies showed its capacity to suppress the immune response within the TME by several specific receptors’ engagement, for example, A1, A2a, A2b, and A3 [66]. To summarize, adenosine acts as a “rheostat” of the immune response, mediating the transition from inflammation to healing.

**Table 2:** Clinical trials on VISTA, CD39, CD73, and CD38

Target	Drug	Combination agent	Phase	Tumor type	Clinical efficacy	PFS	OS	Safety	Clinical trial	Status
VISTA	JNJ-61610588	–	Phase1	Advanced cancer	–	–	–	–	NCT02671955	Terminated
	CI-8993	–	Phase1	Solid tumor	–	–	–	–	NCT04475523	Recruiting
	CA-170	–	Phase 1	Advanced solid tumors or lymphomas	Pharmacokinetics (PK) exposure with a t1/2 of 3.4 h for CA-170	–	–	–	NCT02812875	Completed
		–	Phase 2	Advanced solid tumors or lymphomas	ORR of 30% in Classical Hodgkin lymphoma, CBR of > 85% at a daily dose of 400 mg and PFS of 19.6 weeks in stage 4 non-squamous NSCLC	19.6 weeks	–	–	–	–
CD39	TTX-030	Pembrolizumab Docetaxel Gemcitabine nab-paclitaxel	Phase 1	Solid tumor lymphoma	–	–	–	–	NCT03884556	Active, not recruiting
		Budigalimab Docetaxel mFOLFOX6 Gemcitabine nab-paclitaxel Pembrolizumab	Phase 1	Solid tumor, adult	–	–	–	–	NCT04306900	Active, not recruiting
	SRF617	Gemcitabine Albumin-Bound Paclitaxel Pembrolizumab	Phase 1	Advanced solid tumor	–	–	–	–	NCT04336098	Recruiting
CD73	HLX23	–	Phase 1	Advanced solid tumor	–	–	–	–	NCT04797468	Not yet recruiting
	LY3475070	Pembrolizumab	Phase 1	Advanced cancer	–	–	–	–	NCT04148937	Active, not recruiting
	AK119	AK104	Phase 1	Advanced or metastatic solid tumors	–	–	–	–	NCT04572152	Recruiting
	CPI006	Ciforadenant Pembrolizumab	Phase 1	Advanced solid tumors	1 patient (monotherapy) with metastatic CRPC: substantial reduction in the size of a target lesion after only 5 cycles, sustained at the time of cutoff	–	–	No DLTs reported*	NCT03454451	Recruiting
	Sym024	Sym021	Phase 1	Metastatic cancer solid tumor	–	–	–	–	NCT04672434	Recruiting
	NZV930	KAZ954 PDR001 NIR178	Early Phase 1	Solid tumors	–	–	–	–	NCT04237649	Recruiting
		PDR001 NIR178	Phase 1	NSCLC TNBC PDAC MSS colorectal cancer ovarian cancer RCC mCRPC	–	–	–	–	NCT03549000	Recruiting
	MEDI9447 (Oleclumab)	Osimertinib AZD4635	Phase 1 Phase 2	Carcinoma, NSCLC	–	–	–	–	NCT03381274	Active, not recruiting
		Durvalumab	Phase 1	Muscle-invasive bladder cancer	–	–	–	–	NCT03773666	Active, not recruiting
		Paclitaxel Carboplatin MEDI4736	Phase 1 Phase 2	TNBC	–	–	–	–	NCT03616886	Active, not recruiting
		Durvalumab Tremelimumab MEDI 0562	Phase 2	Ovarian cancer	–	–	–	–	NCT03267589	Completed
		AZD4635 Durvalumab	Phase 2	Prostate cancer mCRPC	Experimental group (*n* = 21): ORR 0.0%; positive control group (*n* = 20): ORR 5.0%*	rPFS 11.1 months for experimental group against 8.8 months for positive control group	Experimental group: NA; Positive control group: 10.72 months	Serious adverse events were 8/30 for experimental group, and 6/29 for control group	NCT04089553	Active, not recruiting
		Durvalumab	Phase 2	Luminal B	–	–	–	–	NCT03875573	Recruiting
		Durvalumab Monalizumab	Phase 2	Stage III NSCLC	–	–	–	–	NCT03822351	Active, not recruiting
CD38	CID-103	–	Phase 1	Multiple myeloma	–	–	–	–	NCT04758767	Recruiting
	Daratumumab	–	Phase 2	Multiple myeloma	–	–	–	–	NCT04656951	Recruiting
		–	Phase 1 Phase 2	GBM	–	–	–	–	NCT04922723	Not yet recruiting
		–	Phase 2	Multiple myeloma in relapse	–	–	–	–	NCT03697629	Completed
		–	Phase 2	Plasma cell myeloma	–	–	–	No report	NCT02944565	Completed
			Phase 2	Relapsed or refractory natural killer/T cell lymphoma	ORR 25.0%, CR 3.1%*	53.0 days	141.0 days	Serious adverse events: 17/32	NCT02927925	Completed
		–	Phase 2	Myeloma multiple	–	–	–	–	NCT03992170	Recruiting
		–	Phase 2	Monoclonal gammopathy smoldering multiple myeloma	–	–	–	–	NCT03236428	Active, not recruiting
		–	Phase 4	Multiple myeloma	–	–	–	–	NCT03768960	Active, not recruiting
		–	Phase 2	Refractory T cell lymphoma relapsed T cell lymphoma	–	–	–	–	NCT04251065	Active, not recruiting
		Nivolumab Cyclophosphamide	Phase 2	Myeloma	–	–	–	–	NCT03184194	Active, not recruiting
		–	Phase 2	Multiple myeloma	–	–	–	–	NCT04230031	Withdraw
		Thalidomide and Dexamethasone	Phase 2	Relapse and/or refractory myeloma	–		–	–	NCT03143036	Unknown
		Velcade, melphalan, and prednisone	Phase 3	Multiple myeloma	The Kaplan–Meier estimate of the 36-month rate of OS was 78.0% (95% CI 73.2–82.0) in the D-VMP group and 67.9% (62.6–72.6) in the VMP group. PFS remained prominently improved for the D-VMP group (HR 0·42 [0·34–0·51]; p < 0·0001)*	–	–	Respiratory infections (54 of 278 patients had upper respiratory tract infections; 42 had bronchitis, and 34 had viral upper respiratory tract infections), cough (34), and diarrhea (28)	NCT02195479 (ALCYONE)	Active, not recruiting
		Lenalidomide and Dexamethasone	Phase 3	Multiple myeloma	mPFS was not reached (95% CI 54.8–not reached) in the patients with Rd compared with 34.4 months (29.6–39.2) in the control group*	NA;34.4 months*	NA	Serious adverse events occurred in 281 (77%) patients with DRd and 257 (70%) patients with Rd. Thirteen (4%) patients with DRd and ten (3%) patients with Rd underwent TRDs	NCT02252172 (MAIA)	Active, not recruiting
		Bortezomib, Thalidomide, and Dexamethasone	Phase 3	Multiple myeloma	157 of 543 patients in the D-VTd group and 110 of 542 patients in the VTd group had achieved a stringent CR; CR was 211 vs 141, and 346 of 543 versus 236 of 542 achieved minimal residual disease negativity*	–	–	46 deaths in the study were observed, and the most common grade 3 or 4 adverse events were neutropenia, lymphopenia, and stomatitis	NCT02541383 (VELCADE)	Active, not recruiting
	MOR03087 (MOR202)	Dexamethasone Pomalidomide Lenalidomide	Phase 1 Phase 2	Multiple myeloma	ORR 0.0% for MOR03087 Biweekly Dose Escalation and MOR03087 Weekly Dose Escalation, 27.8% for MOR03087 Plus Dexamethasone, 47.6% for MOR03087 Plus Pomalidomide + Dexamethasone, 64.7% for MOR03087 Plus Lenalidomide + Dexamethasone	In the given order, 1.1, 2.1, 8.4, 15.9, 26.7(months)	–	In the given order, serious adverse events: 13/31, 4/4, 7/18, 20/21, 14/17	NCT01421186	Completed
	Isatuximab	–	Phase 2	Relapsed multiple myeloma refractory multiple myeloma	–	–	–	–	NCT04802031	Withdraw
		Lenalidomide, Bortezomib, and Dexamethasone	Phase 3	Transplant-eligible NDMM	Minimal residual disease (MRD) negative rate after induction therapy was 35.6% in the RVd group and 50.1% in the isatuximab–RVD group. The CR rate after induction therapy was 24.2% in the isatuximab–RVD group and 21.6% in the RVd group	–	–	At least one AE of grade 3 or higher occurred in 63.6% of patients in the isatuximab–RVD group and 61.3% in the RVd group, respectively. The incidence of serious AEs was 34.8% and 36.3%	GMMG-HD7	–
		Carfilzomib and Dexamethasone	Phase 3	Relapsed multiple myeloma	mPFS was not reached in the isatuximab group compared with 19.15 months in the control group, with an HR of 0.53*	–	–	TEAEs of grade 3 or worse occurred in 136 of 177 patients in the isatuximab group versus 82 of 122 in the control group, serious TEAEs occurred in 105 versus 70 patients, and deaths were reported in six versus four patients	NCT03275285	Active, not recruiting
		Pomalidomide and Dexamethasone	Phase 3	Relapsed and refractory multiple myeloma	mPFS was 11.5 months (95% CI 8.9–13.9) in the isatuximab group, compared with 65 months (4.5–8.3) in the control group	11.5 months; 65 months*	–	Infusion reactions (56), upper respiratory tract infections (69), and diarrhea (68). Serious adverse events were reported in 12 patients (8%) in the isatuximab group and 14 (9%) in the control group	NCT02990338	Active, not recruiting
		Dexamethasone	Phase 2	Relapsed multiple myeloma	–	–	–	–	NCT04965155	Active, not recruiting
		–	Phase 2	Multiple myeloma	–	–	–	–	NCT04786028	Recruiting
		CellProtect	Phase 2	Multiple myeloma	–	–	–	–	NCT04558931	Recruiting
		–	Phase 2	Smoldering plasma cell myeloma	–	–	–	–	NCT02960555	Recruiting
		Bortezomib Dexamethasone	Phase 1	Multiple myeloma	–	–	–	–	NCT04912427	Recruiting
		–	Phase 1	Plasma cell myeloma	MR 3.1%, SD 53.1%	1.6	10.7	16 (50.0%) patients had Grade ≥ 3 TEAE*	NCT02514668	Completed
		–	Phase 1 Phase 2	Multiple myeloma	ORR 36.4%*, CBR 54.5%	4.7	–	No DLTs reported*. TEAEs of grade ≥ 3 included pneumonia in two patients, and intervertebral discitis, lung infection, disseminated intravascular coagulation, seizure, thrombotic cerebral infarction, ileus, and synovial cyst in one patient each	NCT02812706	Active, not recruiting
		Cemiplimab	Phase 2	Natural killer/T cell lymphoma relapsed natural killer/T cell lymphoma refractory natural killer/T cell lymphoma	–	–	–	–	NCT04763616	Recruiting
		Carfilzomib Pomalidomide	Phase 2	Recurrent plasma cell myeloma refractory plasma cell myeloma	–	–	–	–	NCT04850599	Recruiting
		–	Phase 1	Multiple myeloma	–	–	–	–	NCT03733717	Active, not recruiting
	ISB 1342	–	Phase 1	Relapsed/refractory multiple myeloma	–	–	–	–	NCT03309111	Recruiting
	GEN3014	–	Phase 1 Phase 2	Multiple myeloma	–	–	–	–	NCT04824794	Recruiting

*Primary endpoint; NSCLC, non-small -cell lung cancer; TNBC, triple-negative breast cancer; MSS, microsatellite stable; PDAC, pancreatic ductal adenocarcinoma; RCC, renal cell carcinoma; mCRPC, metastatic castration-resistant prostate cancer; GBM, glioblastoma

However, Otto Warburg pointed out that the proliferation of cancer cells was linked to energy generation mainly through the anaerobic breakdown of glucose instead of the oxidative breakdown of pyruvate in normal tissue cells [67]. Therefore, the tumor microenvironment might be a perfect place for adenosine formation adenosine probably engages in the immune escape, which has been confirmed by a recent study [68].

Adenosine is generated in the TME through the coordinated activity of the ectonucleotidases CD39 (ENTPD-1) and CD73 (5’-NT or NT5E), in which the extracellular adenosine triphosphate (ATP) [69], an inflammation-inducing factor, is converted to adenosine. Given that, CD39 and CD73 became essential targets for cancer therapy. Besides the CD39-CD73 chain, the CD38-NPP1-CD73 chain utilizes nicotinamide adenine dinucleotide (NAD) as the precursor via intermediates ADP-ribose (ADPR) and cADPR to generate AMP. Several studies have identified the functional role CD38 played in the reaction chain and targeted CD38 for therapy [70].

CD39, also known as ENTPD-1, is the rate-limiting ectoenzyme in extracellular ATP hydrolysis [71]. Moreover, CD73 utilizes the product, 5′-adenosine monophosphate (5′-AMP), to generate extracellular adenosine [72]. The expression level of CD39 is upregulated under the stimulus from hypoxia-induced factor (HIF)-1, transforming growth factor (TGF)-β, and epithelial-to-mesenchymal-transition (EMT). CD39 expression could also be increased by T cell exhaustion and chronic inflammatory factors, such as IL-6 and TNF-α. CD39 is found to be broadly produced on B cells, NK cells, DCs, monocytes, macrophages, Tregs [73], also been seen on fibroblasts, myeloid cells, vascular endothelial cells, Tregs, and tumor-specific T effector cells in TME [71]. The wide distribution of CD39 indicates its abundant biological functions. (1) NLRP3 inflammasome is activated by extracellular ATP through the P2X7 receptor to induce pyroptosis [71], and CD39 can suppress the reaction by downregulating the ATP level. (2) The release of ATP by some dying tumor cells, as well as calreticulin exposure and high-mobility group protein 1 (HMGB1) secretion, are thought to be the hallmarks of immunogenic cell death, and it has been verified that the death of transformed cells often elicits highly effective anti-tumor immune responses [74]. CD39 could deplete ATP, thus cutting off the activation of macrophages, which is critical for dying tumor cells’ immunogenicity. (3) It was shown that the chemotaxis of macrophages lies on the gradient of extracellular ATP concentrations [75]. Thus, the gradient will be blocked if adding excessive exogenous ATP or using soluble CD39-like apyrase. Since increasing extracellular ATP levels also activated P2X4 and P2X7 receptors, the activation would trigger calcium signaling among neighboring macrophages [76] and eventually promote macrophage phagocytosis. It was thought that CD39 also plays a role in suppressing phagocytosis.

CD73, an ecto-5′-nucleotidase, is a cell surface glycosylphosphatidylinositol-anchored glycoprotein. CD73 has widely been the surface marker of several cell types, such as endothelial cells, subtypes of lymphocytes [77], stromal cells, and tumor cells. In tumor-infiltrating NK cells, CD73 is transported from intracellular vesicles to the cell surface and the extracellular matrix via actin polymerization–dependent exocytosis with the engagement of 4-1BBL on tumor cells [78]. Following the focal radiotherapy, inflammation and tissue damage induce the increased level of adenosine and CD73, and CD73 blockade promotes DC infiltration of tumors and tumor rejection which facilitates IFN-I inducing the infiltration of tumor [79]. Metformin has long been a potent drug for type 2 diabetes, and it has also been recently reported to modulate CD73/CD39 expression on MDSCs through activation of the AMPKα pathway and inhibition of the HIF-1α pathway, enhancing the immune response [69].

CD38 belongs to the ribosyl cyclase family and is widely expressed on the surface of non-hematopoietic cells and several immune cells. In addition to the extracellular adenosine, NAD+ is another critical factor for immune escape. It has been confirmed to participate in a series of reactions, like cell proliferation, leucocyte differentiation, as well as function [80–82]. However, CD38 is an ectoenzyme and transmits NAD+ to ADP-ribose (ADPR) and cADPR. It has been proven to significantly affect intracellular Ca2+, cell adhesion, and signal transduction [83]. Th1 and Th17 cells were potent anti-tumor T cells for their heightened effector function and prolonged persistence. They need a functional NAD+-Sirt1 Axis to exert an anti-tumor response, which research proved that the anti-CD38 antibody could enhance the inhibition of tumor [80].

Overall, the extracellular ATP activated by inflammation is an essential inducer of the immune response, and the adenosine suppresses the reaction. Till now, we knew CD39 and CD73 worked together to mediate the transformation of extracellular ATP to adenosine, which took up most of the synthesis of extracellular adenosine. CD38 is a main bypass of the adenosine synthesis, and the precursor used, NAD+, also plays a vital role in immunoregulation. All in all, a deeper understanding of the three enzymes is needed to promote immunotherapy.

### Clinical trials on CD73, CD39, and CD38 (Table 2)

There are 425 clinical trials of CD38 ongoing or completed on Clinicaltrials.gov. Multiple myeloma (MM) is the second most common hematologic tumor, until anti-CD38 antibodies Daratumumab and Isatuximab have been approved for treating multiple myeloma. Daratumumab induces cell death through complement-dependent cytotoxicity (CDC), antibody-dependent cell-mediated cytotoxicity (ADCC), antibody-dependent cellular phagocytosis (ADCP), induction of apoptosis, and modulation of CD38 enzyme activities. Daratumumab may also exhaust CD38+ immune regulatory cells and promote T cell expansion. Thus, it accelerates the exhaustion of tumor cells. It was approved by FDA in 2015 and applied in treating multiple myeloma [77].

- In the phase III study of MAIA (NCT02252172), Scientists enrolled 737 patients with MM not appropriate for NDMM transplantation and divided them into two groups: one group receiving Lenalidomide and Dexamethasone (Rd, 369), the other receiving Daratumumab, Lenalidomide, and Dexamethasone (DRd, 368). At a median follow-up of 56.2 months (IQR 52.7–59.9), mPFS was not reached (95% CI 54.8–not reached) in the patients with Rd compared with 34.4 months (29.6–39.2) in the control group (HR 0.53 [95% CI 0.43–0.66]; p < 0.0001), the two groups did not reach mOS either (HR = 0.68, *p* = 0.0013). The most common (> 15%) grade 3 or higher treatment-emergent adverse events included neutropenia, pneumonia, anemia, and lymphopenia. Serious adverse events occurred in 281 (77%) patients with DRd and 257 (70%) patients with Rd. Thirteen (4%) patients with DRd and ten (3%) patients with Rd underwent TRDs [84].
- Another phase III study is about ALCYONE (NCT02195479). Seven hundred and six patients with MM were enrolled, 356 received Velcade, Melphalan, and Prednisone (VMP), and the rest received Daratumumab, Velcade, Melphalan, and Prednisone (D-VMP). The HR for death in the D-VMP group compared with the VMP group was 0.60 (95% CI 0.46–0.80; *p* = 0.0003). The Kaplan–Meier estimate of the 36-month rate of OS was 78.0% (95% CI 73.2–82.0) in the D-VMP group and 67.9% (62.6–72.6) in the VMP group. PFS remained prominently improved for the D-VMP group (HR 0·42 [0·34–0·51]; p < 0·0001). The most frequent adverse events during maintenance of Daratumumab monotherapy in patients in the D-VMP group were respiratory infections (54 of 278 patients had upper respiratory tract infections; 42 had bronchitis, and 34 had viral upper respiratory tract infections), cough (34), and diarrhea (28) [85].

MAIA and ALCYONE put Daratumumab into the first-line treatment regimen for MM, and D-VMP and DRd became the preferred treatment regimen for patients unsuitable for NDMM transplantation.

- CASSIOPEIA is a phase III study showing the clinical benefit of Daratumumab combined with bortezomib, thalidomide, and dexamethasone (VTd) in transplant-eligible patients with MM (NCT02541383). Totally 1085 patients were enrolled and randomly assigned D-VTd (*n* = 543) or VTd (*n* = 542). The result showed that 100 days after transplantation, 157 of 543 patients in the D-VTd group and 110 of 542 patients in the VTd group had achieved a stringent CR (odds ratio 1.60, 95% CI 1.21–2.12, *p* = 0.0010). CR was 211 vs 141, and 346 of 543 versus 236 of 542 achieved minimal residual disease negativity (*p* < 0·0001). The two groups did not achieve mPFS either. Forty-six deaths in the study were observed (14 vs 32, 0.43, 95% CI 0.23–0.80), and the most common grade 3 or 4 adverse events were neutropenia, lymphopenia, and stomatitis [86]. The CASSIOPEIA study showed that compared with VTd, D-VTD showed a better and long-lasting efficacy. At the same time, the safety did not differ, making it an excellent benefit for MM patients capable of transplantation.

Isatuximab is another CD38 monoclonal antibody that also targets MM. It has an entirely different antigen-binding epitope compared to Daratumumab, which can almost completely inhibit the enzymatic activity of CD38 and directly cause the apoptosis of tumor cells through FcγR-mediated cross-linking. It has a better scavenging effect on CD38-positive NK cells and Treg cells. Recent studies have stepped into phase III trials (NCT02990338), showing that Isatuximab extended patients’ PFS [87].

- In a phase III study (NCT02990338), scientists enrolled 307 patients with relapsed and refractory multiple myeloma and then randomly assigned them to treatment: 154 to Isatuximab–pomalidomide–dexamethasone and 153 to pomalidomide–dexamethasone. At a median follow-up of 11.6 months (IQR 10.1–13.9), mPFS was 11.5 months (95% CI 8.9–13.9) in the Isatuximab group, compared with 65 months (4.5–8.3) in the control group (HR 0.596, 95% CI 0.44–0.81; *p* = 0.001). The most frequent treatment-emergent adverse events were infusion reactions (56), upper respiratory tract infections (69), and diarrhea (68). Serious adverse events were reported in 12 patients (8%) in the Isatuximab group and 14 (9%) in the control group. One case in the Isatuximab group (sepsis) and two in the control group (pneumonia and urinary tract infection) reported deaths [87].
- IKEMA is a phase III study showing the efficacy of Isatuximab plus carfilzomib–dexamethasone versus carfilzomib–dexamethasone in patients with relapsed multiple myeloma (NCT03275285). Three hundred and two patients were enrolled: 179 were randomly assigned to the Isatuximab group and 123 to the control group. The result showed that mPFS was not reached in the Isatuximab group compared with 19.15 months (95% CI 15.77–not reached) in the control group, with an HR of 0.53 (99% CI 0.32–0.89; one-sided *p* = 0.0007). TEAEs of grade 3 or worse occurred in 136 of 177 patients in the Isatuximab group versus 82 of 122 in the control group, serious TEAEs occurred in 105 versus 70 patients, and deaths were reported in six versus four patients [88].
- GMMG-HD7 is a phase III study showing the clinical benefit of Isatuximab in combination with lenalidomide, bortezomib, and dexamethasone (isatuximab–RVD) in patients with transplant-eligible NDMM. The minimal residual disease (MRD) negative rate after induction therapy was 35.6% in the RVd group and 50.1% in the isatuximab–RVD group. The CR rate after induction therapy was 24.2% in the isatuximab–RVD group and 21.6% in the RVd group. Regarding safety, at least one AE of grade 3 or higher occurred in 63.6% of patients in the isatuximab–RVD group and 61.3% in the RVd group, respectively. The incidence of serious AEs was 34.8% and 36.3%, respectively, and a comparable number of patients discontinued induction therapy because of AEs in the two groups. Isatuximab–RVd combination regimen can be used as the optimal regimen for NDMM patients undergoing transplantation, which has a good application prospect.

CD38 antibody has also been applied to lymphoma.

- In the study (NCT02927925), 32 participants were enrolled. Everyone received Daratumumab 16 mg/kg by IV infusion to assess Daratumumab's clinical efficacy and safety in relapsed or refractory natural killer/T cell lymphomas (NKTCL). Fortunately, the primary outcome ORR of Daratumumab monotherapy was 25.0% of the total 32 Asian patients, no patient achieved CR, six patients had SD and 14 cases underwent PD, and PFS reached 53.0 days, while OS comes to 141.0 days. The median duration of response of the eight responders was 55.0 days [89]. There are 17 out of 32 participants who suffered severe adverse events, including pyrexia (5 cases), thrombocytopenia (3 cases), septic shock (3 cases), etc.

Till now, all clinical trials on lymphoma have reached few results. Some even terminated due to unsatisfactory outcomes [90, 91] (NCT02999633, NCT02413489). All in all, studies on lymphoma are not recommended until breakthroughs occur.

- There are 19 clinical trials on CD73 on Clinicaltrials.gov, among which MEDI9447 (Oleclumab) is a human monoclonal antibody targeting CD73. In a phase II study (NCT04089553), researchers applied it as a combination therapy with AZD4635 or Durvalumab in patients with mCRPC who progressed on standard treatments. Among the 59 participants, 29 (Module 1) received monotherapy of AZD4635. The rest 30 patients (Module 2) received combination therapy of AZD4635 and Oleclumab. ORR is 5.0% in Module 1 and 0 in Module 2, respectively. The percentage of participants with prostate-specific antigen (PSA) is 3.6% and 3.3%, respectively. The percentage of participants with PFS at six months is 8.8% and 11.1% in Modules 1 and 2, respectively. The result indicated that Oleclumab has few benefits for patients with prostate cancer, and further studies are needed.
- In another study (NCT04672434), researchers dug into Sym024, a newly developed anti-CD73 antibody, exploring Sym024’s safety and tolerance as monotherapy or in combination with Sym021 in patients suffering from solid tumor malignancies. However, no results have been posted either.
- And TTX-030 is an uncompetitive allosteric inhibitor of CD39. By using an endpoint Malachite Green-based assay that detects the release of free phosphate (Pi), TTX-030 inhibited rhCD39-ECD ATPase activity with an IC50 of 0.20 ± 0.06 nM with 55% maximal inhibition (NCT03884556) [92]. In another study (NCT04306900), researchers combined TTX-030 with immunotherapy and chemotherapy like Budigalimab, Docetaxel, or Gemcitabine in patients suffering from solid tumors. The research was expected to improve the accumulation of pro-inflammatory ATP and immunosuppressive adenosine reduction, but the result has not been published.

ES014 is an anti-CD39/TGF-β bispecific antibody; a relevant study (NCT05381935) has been conducted on patients with advanced solid tumors, but the result has not come out yet.

- CD39 and CD73, two ectonucleotidases, usually work together to upregulate the level of intercellular adenosine. Adenosine attaches to the A2A receptor to convey a signal repressing T cell activation [93]. Researchers tend to obstruct the two pathways for a better result. Although few studies are registered on Clinicaltrials.gov, we are inspired to see that many preclinical studies have taken place. In one study, researchers applied IPH5201 and IPH5301, which targeted CD39 and CD73, respectively, to mice with melanoma or colorectal cancer [94]. The result showed a decline in Ado accumulation and a limitation of Ado-mediated T cell inhibition. Overall, combinatory drug use needs more and more profound studies.

## LAG-3

### Structure and function

LAG-3 is identified as a type I transmembrane protein. It is mainly found on activated T cells, NK cells, B cells, and plasmacytoid dendritic cells [95]. It comprises four extracellular immunoglobulin (Ig)-like domains (D1–D4) that share 20% amino acid homology with CD4, and the similarity lies in the proximity of LAG-3 to CD4 on the human chromosome 12. Opposite to the semblable in the extracellular regions, intracellular parts of LAG-3 and CD4 show no noticeable similarity. For example, LAG-3 lacks the cysteine motif required to link with lymphocyte-specific protein tyrosine kinase (Lck) and the palmitoylation site observed in CD4 [96, 97]. The construction of the genomic parts of CD4 and LAG-3 obtaining exons encoding their extracellular regions is similar. Still, the genomic building receiving exons encoding their intracellular regions varies, indicating an early divergence of the two genes during evolution [98]. Now, we found that the LAG-3 cytoplasmic tail contains three conserved motifs. And it mediates intracellular negative signal transduction: (1) a potentially phosphorylatable serine (S484), (2) a KIEELE motif, and (3) a glutamate-proline dipeptide multiple repeat motif (EP motif). S484 has been found its correlate with IL-2 production [99]. Lysine residue (K468) found in KIEELE was required for LAG-3 downstream signaling. The EP motif was vital in promoting colocalization of LAG-3 with CD3, CD4, and CD8 within lipid rafts through interacting with LAP protein (LAG-3 associated protein) [100].

Since LAG-3 is structurally like the CD4 co-receptor, it links to the MHC class II complex with a stronger affinity than CD4. LAG-3 also affects the activity of CD8+ T cells and NK cells, which interact with the MHC class II complex. The fact has pointed to alternate ligands for Lag-3 [101]. The DC-SIGN family member LSECtin and the liver-secreted protein FGL1 have now been identified as ligands of LAG-3. Besides, studies showed that the silence of LAG-3 and PD-1 independently has little effect on autoimmunity [102]. At the same time, the double-knockout of LAG-3 and PD-1 in the mice model resulted in multi-organ lymphocytic infiltration, indicating a closer relationship between LAG-3 and PD-1 in regulating T cell viability [98].

CD4+ T cells-expressed LAG-3 binds to the MHC class II complex on antigen-presenting cells, while CD8+ T cells-expressed and NK cells-expressed LAG-3 links to the LSECtin on tumor cells or liver cells. The unique KIEELE motif on the cytoplasmic tail of LAG-3 is critical for its immune suppressive function [101]. Studies showed that the deletion of KIEELE completely abrogates LAG-3 function on CD4 T cells [103]. Since LAG-3 has a significantly higher affinity with MHC-II than CD4, it is no surprise that LAG-3-Ig fusion proteins act as competitors in CD4/MHC class II-dependent cellular adhesion assays downregulate T cell activation, cytotoxicity, and cytokine production [103]. The cytoplasmic domain of LAG-3 would transmit inhibitory signals and inhibit CD4 T cell activation once LAG-3 is tied to MHC-II [104, 105]. Besides, the bination of LAG-3 to MHC class II complex also dampens Th cell response. Overall, the high-affinity LAG-3: MHC class II bination was believed to be the primary mechanism of the high inhibitory competence of LAG-3. It works through the competition with CD4: MHC-II binding in early studies, while along with the novel identification of additional ligands, a consensus is under controversy now.

### Clinical trials on LAG-3 (Table 3)

**Table 3:** Clinical trials on LAG-3

Target	Drug	Combination agent	Phase	Tumor type	Clinical efficacy	PFS (m)	OS (m)	Safety	Clinical trial	Status
LAG-3	BMS 986,016(Relatlimab)	Nivolumab	Phase 2/3	Melanoma	Researchers provided data on PFS*, ORR of the Relatlimab–Nivolumab group and the Nivolumab group was 43% and 33%	10.12 for group: Relatlimab + Nivolumab, and 4.63 for group: Nivolumab*	NR for group: Relatlimab + Nivolumab, and 34 for group: Nivolumab	Serious adverse events were 108/355 for Group A: Relatlimab + Nivolumab, and 119/359 for Nivolumab	NCT03470922 (RELATIVITY-047)	Active, not recruiting
		Nivolumab	Phase 1	Cancer	–	–	–	–	NCT02966548	Active, not recruiting
		BMS-936558	Phase 1	Hematologic neoplasms	–	–	–	–	NCT02061761	Active, not recruiting
		Nivolumab	Phase 2	Melanoma	–	–	–	–	NCT03743766	Recruiting
		Nivolumab, BMS-986213	Phase 1	Neoplasms by site	–	–	–	–	NCT01968109	Active, not recruiting
			Phase 2							
		Nivolumab	Phase 2	Metastatic uveal melanoma	–	–	–	–	NCT04552223	Recruiting
		Nivolumab	Phase 1	Glioblastoma	–	–	–	–	NCT03493932	Active, not recruiting
		Nivolumab	Phase 2	Chordoma, locally advanced chordoma, metastatic chordoma, unresectable chordoma	–	–	–	–	NCT03623854	Recruiting
		Anti-PD-1	Phase 1	Glioblastoma	–	–	–	–	NCT02658981	Active, not recruiting
		Anti-CD137		Gliosarcoma, recurrent brain neoplasm						
		Elotuzumab, pomalidomide, dexamethasone	Phase 1	Multiple myeloma	–	–	–	–	NCT04150965	Recruiting
		Azacitidine Injection	Phase 2	Acute myeloid leukemia	–	–	–	–	NCT04913922	Recruiting
		Nivolumab	Phase 3	Melanoma	–	–	–	–	NCT05002569	Not yet recruiting
		Nivolumab	Phase 2	Hepatocellular carcinoma, hepatoma	–	–	–	–	NCT04567615	Recruiting
		Nivolumab	Phase 1	Various advanced cancers	ORR 0 for HPV-positive SCCHN *	3.81	8.84	Serious adverse events included one case for angina pectoris, pathological fracture, pleural effusion, pulmonary hemorrhage, stridor each, two cases for malignant neoplasm, and three cases for dyspnea*	NCT02488759	Active, not recruiting
		Nivolumab	Phase 1	Advanced cancer	–	–	–	–	NCT03459222	Recruiting
		Nivolumab	Phase 2	Advanced cancer	–	–	–	–	NCT02996110	Recruiting
		Nivolumab	Phase 1	Hepatocellular carcinoma	–	–	–	–	NCT04658147	Recruiting
		Nivolumab + Ipilimumab	Phase 2	HNSCC	–	–	–	–	NCT04326257	Recruiting
		Nivolumab	Phase 2	MSS colorectal adenocarcinomas	–	–	–	–	NCT03642067	Recruiting
		Nivolumab	Phase 2	Refractory MSI-H solid tumors prior to PD-(L) 1 therapy	–	–	–	–	NCT03607890	Recruiting
		Nivolumab	Phase 2	HNSCC	–	–	–	–	NCT04080804	Recruiting
		Nivolumab	Phase 2	Advanced gastric cancer	–	–	–	–	NCT02935634	Active, not recruiting
		Nivolumab, Carboplatin, Paclitaxel, Radiation	Phase 1	Gastric cancer, esophageal cancer, gastroesophageal cancer	–	–	–	–	NCT03044613	Active, not recruiting
	IMP321 (Eftilagimod Alpha)	Gemcitabine	Phase 1	Pancreatic neoplasms	–	–	–	–	NCT00732082	Terminated
		Pembrolizumab	Phase 1	Stage IV melanoma, stage III melanoma	1/18 CR	–	–	No DLTs reported*	NCT02676869	Completed
		Paclitaxel, Placebo	Phase 2	Adenocarcinoma breast stage IV	–	–	–	–	NCT02614833	Active, not recruiting
		–	Phase 1	Advanced RCC	7 of 8 patients treated with the higher doses of IM321 underwent SD at three months. 3 of 11 in the lower dose group did	–	–	No clinically significant local or systemic treatment-related adverse events were recorded. Along with the 195 adverse events, 20 (10%) were reported to be related to IMP321 and were grade 1 local reactions*	NCT00351949 (P003)	Completed
		Avelumab	Phase 1	Solid tumors, peritoneal carcinomatosis	ORR 17% and DCR 33% with 1/6 PR, 1/6 SD, and 4/6 PD	–	–	No DLTs reported*	NCT03252938	Recruiting
		Paclitaxel	Phase 1	Metastatic breast cancer	ORR 50% and 1/10 PD	–	–	Six grade 3 adverse events were recorded, including asthenia (3 cases), neuropathy, allergic reaction, and neutropenia*	NCT00349934	Completed
		Pembrolizumab	Phase 2	NSCLC, HNSCC	ORR 47%* and DCR 82% with 8/17 PR and 6/17 SD	–	–	Most common toxicities included cough (31%), fatigue (19%), and diarrhea (15%)	NCT03625323	Recruiting
		Pembrolizumab	Phase 2	HNSCC	–	–	–	–	NCT04811027	Not yet recruiting
		Paclitaxel	Phase 1	Metastatic breast cancer	–	–	–	–	NCT04252768	Not yet recruiting
		Immunological peptides and immunological adjuvants	Phase 1	Melanoma	–	–	–	–	NCT00365937	Terminated
		HLA-A2 peptides	Phase 2							
		Montanide ISA-51								
		Melan-A VLP vaccine, cyclophosphamide, fludarabine phosphate	Phase 1	Melanoma	–	–	–	Grade 3–4 treatment-related adverse events included anemia (16.6%), leucopenia (100%), thrombocytopenia (33%), febrile neutropenia (grade 3) (50%), CD4 lymphopenia (≥ grade 3) (100%) *	NCT00324623	Completed
	Sym022	–	Phase 1	Metastatic cancer, solid tumor, lymphoma	33.3% SD in group: Dose Level 3	–	–	Serious adverse events were 1/3 in Dose Level 3(chest pain) and 2/6 in Dose Level 4 (gastrointestinal hemorrhage, sepsis, lipase increased, and tumor pain) *	NCT03489369	Completed
		Sym021	Phase 1	Metastatic cancer, solid tumor	–	–	–	–	NCT04641871	Active, not recruiting
		Sym021	Phase 1	Metastatic cancer, solid tumor, lymphoma	–	–	–	–	NCT03311412	Recruiting
	INCAGN02385	–	Phase 1	Cervical cancer, MSI-high endometrial cancer	–	–	–	–	NCT03538028	Completed
		INCAGN02390, INCMGA00012	Phase 1/2	Melanoma	–	–	–	–	NCT04370704	Recruiting
	REGN3767	–	Early Phase 1	Large B cell lymphoma, DLBCL	–	–	–	–	NCT04566978	Recruiting
		Cemiplimab	Phase 1	Malignancies	Monotherapy group: ORR 0% and DCR 48% with 12 SD Combination group: ORR 5% and DCR was 31% with 2 PR and 11 SD 2/12 PR and 6 SD in the group crossed over from monotherapy to the combination	–	–	1/67 DLT in the combination group (G4 CK elevation + G3 myasthenic syndrome + G1 elevation of troponin*	NCT03005782	Recruiting
		Cemiplimab	Phase 1/2	Metastatic solid tumor	–	–	–	–	NCT04706715	Not yet recruiting
	RO7247669	–	Phase 1	Solid tumors, metastatic melanoma, NSCLC, esophageal squamous cell carcinoma	–	–	–	–	NCT04140500	Recruiting
		RO7121661	Phase 2	Advanced or metastatic esophageal squamous cell carcinoma	–	–	–	–	NCT04785820	Recruiting
		Atezolizumab	Phase 1	Advanced liver cancers	–	–	–	–	NCT04524871	Recruiting
	BI 754,091	Anti-PD-1	Phase1	Advanced or metastatic solid tumors	–	–	–	21/321 DLTs, particularly infusion-related reactions (*n* = 6). Serious AEs: 77/321 (27%): pleural effusion (*n* = 6), deep venous thrombosis (*n* = 4), cardiac tamponade (*n* = 1), and acute kidney injury (*n* = 1)	NCT03156114	Active, not recruiting
		Anti-PD-1	Phase1						NCT03433898	Active, not recruiting
		Anti-PD-1	Phase1						NCT03780725	Terminated
		Anti-PD-1	Phase2						NCT03697304	Active, not recruiting
	EMB-02	–	Phase 1	Advanced solid tumors					NCT04618393	Recruiting
	LAG525	PDR001	Phase 2	SCLC, gastric adenocarcinoma, esophageal adenocarcinoma, castration-resistant prostate adenocarcinoma, soft tissue sarcoma, ovarian adenocarcinoma, advanced well-differentiated neuroendocrine tumors	ORR 9.3%, DCR for neuroendocrine tumors (86%), diffuse large B cell lymphoma (43%), and small-cell lung cancer (27%)*	2.8	–	11/72 patients had grade 3 or 4 AEs including dyspnea, fatigue, and poor appetite	NCT03365791	Completed
				DLBCL						
		Spartalizumab	Phase 2	Triple-negative breast cancer	ORR for LAG525 + Spartalizumab (5.0), LAG525 + Spartalizumab + Carboplatin (32.4), LAG525 + Carboplatin (17.6)*	LAG525 + Spartalizumab (1.4), LAG525 + Spartalizumab + Carboplatin (4.3), LAG525 + Carboplatin (3.0)	–	Serious adverse events for LAG525 + Spartalizumab (6/19), LAG525 + Spartalizumab + Carboplatin (12/34), LAG525 + Carboplatin (14/34)	NCT03499899	Completed
		PDR001	Phase 1	Advanced solid tumors	11/121 patients in the combination group achieved PR 1 patient had a CR	–	–	DLTs occurred in 4/121 patients including grade 3 and 4 pneumonitis, acute kidney injury, and autoimmune hepatitis*	NCT02460224	Completed
		Spartalizumab	Phase 1	TNBC	–	–	–	–	NCT03742349	Recruiting
		Spartalizumab	Phase 2	Melanoma	–	–	–	–	NCT03484923	Recruiting
	MGD013	Alone or in combination with margetuximab (for patients who had expression of HER2 on their tumors)	Phase1	Advanced or metastatic solid or hematologic malignancies	Dose escalation (*n* = 29): ORR 10% and DCR 55% with 3 confirmed PR, 1 unconfirmed PR, and 13 SD Expansion cohort (*n* = 41): ORR 7%, DCR 59% with 3 PR, and 21 SD	–	–	2/207 DLTs: immune-mediated hepatitis and increased lipase*	NCT03219268	Active, not recruiting
	FS118		Phase1	Advanced solid tumors	–	–	–	–	NCT03440437	Recruiting

*Primary endpoint; NSCLC, non-small-cell lung cancer; SCLC, small-cell lung cancer; HNSCC, head and neck squamous cell carcinoma; MSS, microsatellite stable; UC, urothelial cancer; TNBC, triple-negative breast cancer; DLBCL, diffuse large B cell lymphoma; MSI, microsatellite instability

Recent studies have focused on developing antagonistic mAbs, IMP321 (Eftilagimod Alpha), a soluble dimeric recombinant protein composed of four LAG-3 extracellular domains fused to the Fc portion of human IgG1 (LAG-3-Ig) [106].

- The first phase I trial using the dose-escalated IMP321 as monotherapy was conducted on 24 patients suffering from advanced metastatic renal cell carcinoma in 2006 (NCT00351949, P003). Patients with advanced RCC were treated with escalating doses of IMP321 s.c. Their blood samples were tested to detect human anti-IMP321 antibody formation and determine long-lived CD8 T cell responses. Although no therapy response was reported, 7 of 8 patients treated with the higher doses of IM321 underwent SD at three months. On the contrary, only 3 of 11 in the lower dose group did that (*p* = 0.015). No clinically significant local or systemic treatment-related adverse events were recorded. Along with the 195 adverse events, 20 (10%) were reported to be related to IMP321 and were grade 1 local reactions [107].
- IMP321 has also been combined with first-line chemotherapy and immunotherapies in a series of clinical trials conducted in melanoma (NCT02676869, NCT01308294, NCT00324623), metastatic breast cancer (NCT00349934), and pancreatic neoplasms (NCT00732082). Many researchers have reported promising results of IMP321 in melanoma therapy, among which four studies have been reported on ClinicalTrials.gov.

Besides, the development of antagonistic monoclonal antibodies targeting LAG-3 drew much attention. BMS-986016 (Relatlimab) is a human IgG4 anti-LAG-3 blocking mAb, and multiple studies have proven the synergistic impact of anti-LAG-3 with PD-1/PD-L1-targeted therapies.

- So far, the safety and efficacy of Relatlimab administered alone and in combination with anti-PD-1 monoclonal antibody (Nivolumab, BMS-936558) were explored in advanced solid tumors in a phase I study (NCT02966548), no result reported.
- In another study (NCT02488759), 578 participants were divided into six groups to investigate the safety and efficacy of Nivolumab combination therapy in virus-associated Tumors. in Metastatic Combo C (8 participants), everyone received Nivolumab 240 mg IV over 30 min + Relatlimab 80 mg IV over 60 min administered every two weeks for a maximum of 24 months. ORR is 0 for the 8 participants with HPV-positive SCCHN, PFS is 3.81 months, and OS is 8.84 months. Five out of 8 patients underwent severe adverse events, including one case for angina pectoris, pathological fracture, pleural effusion, pulmonary hemorrhage, stridor, two cases for malignant neoplasm, and three cases for dyspnea. However, the sample capacity is too small. It is insufficient to reach a firm conclusion.

As a critical and a pivotal trial, the up-to-date study RELATIVITY-047 (NCT03470922) suggested that inhibiting both LAG-3 and PD-1 with Relatlimab and Nivolumab (Opdualag, BMS-986213, fixed-dose combination Relatlimab and Nivolumab at a 1:3 ratio) provides a better prognosis than those who only received Nivolumab in patients with previously untreated metastatic or unresectable melanoma, which led to FDA approval on the first anti-LAG-3 antibody Relatlimab for the treatment of melanoma.

- Specifically, the primary endpoint mPFS of the Relatlimab–Nivolumab group was 10.1 months compared with 4.6 months of Nivolumab monotherapy (HR = 0.75). ASCO 2022 updated that the Relatlimab–Nivolumab group did not reach mOS while the mOS of Nivolumab was 34 m (HR = 0.81), which showed that patients treated with Relatlimab–Nivolumab had better survival, but it is not significant. And ORR of the Relatlimab–Nivolumab group and the Nivolumab group was 43% and 33%, respectively. All the subgroup analyses (including PD-L1 and LAG-3 expression, BRAF mutation status, M1c stage, higher tumor burden, higher LDH, etc.) favored Relatlimab–Nivolumab over Nivolumab, and ORR were higher in patients with LAG-3 ≥ 1% and PD-L1 ≥ 1%. Patients in the Relatlimab–Nivolumab group suffered more grade 3 or 4 treatment-related adverse events than those in the Nivolumab group (18.9% to 9.7%). Since the participants in the Relatlimab–Nivolumab group had a longer PFS, the implications of the adverse events occurrence rates need to be clarified. Besides, the treatment-related adverse events among the two groups did not show many differences, including hypothyroidism or thyroiditis, rash, and diarrhea or colitis [108].

LAG-3 is an immune checkpoint of great potential. Relatlimab, an anti-LAG-3 blocking mAb, has been approved for melanoma treatment. In the latest studies, Relatlimab can also be seen in treating NSCLC or gastric cancer. Maybe some inspiring news will come from treatments of other types of tumors soon. Although IMP321 is also expected, it lacks approval for treating melanoma, and more studies must be done.

## IDO-1

### Structure and function

IDO-1 is essential in regulating immune escape. It catalyzes the oxidation of Trp (l-tryptophan) to form N-formyl L-kynurenine, rapidly converted by formamidases to Kyn (kynurenine) [109]. Rocketed levels of Kyn and higher plasma Kyn/Trp ratios are often found in cancer patients at an advanced stage and correlate with poor prognosis [110]. The importance of Trp starvation responses against Kyn accumulation in the TME as the driving force for immunosuppression has been debated for over 20 years [110].

Generally, Trp is a critical amino acid for mammals. The tryptophan pool in tumor microenvironments will be restricted in response to the abnormal activation of IDO-1, leading to decreased T cells [111]. Besides, the reduced concentration of local Trp triggered the activation of the general control nonderepressible 2 kinases (GCN2) [110]. Kyn is an AhR (Aryl hydrocarbon Receptor) ligand. The interaction leads to the suppressed secretion of IFN-I and activation of NF-κB signaling [112]. Kyn also converted naive CD4+ T cells into Foxp3+ Treg cells and the Trp starvation [109]. Besides its immediate effect on AhR, in vitro Kyn is slowly transmitted from non-enzymatically to byproducts that serve as high-affinity (sub-nM) AhR agonists. In addition, the downstream metabolites of the Trp catabolism, 3′OH-kynurenine, and 3′OH-anthranilic acid, also act as AhR ligands [110]. As the intermediate product of the KP (kynurenine pathway), Kyn is further metabolized through the KP to quinolinic acid. And the latter is switched to NaMN through the enzyme quinolate phosphoribosyltransferase (QPRT). Then, it ultimately switched to NAD+ via the Preiss-Handler pathway, thus joining the NAD+ immunoregulation pathway [113].

### Clinical trials on IDO-1 (Table 4)

**Table 4:** Clinical trials on IDO-1 and CD27

Target	Drug	Combination agent	Phase	Tumor type	Clinical efficacy	PFS	OS	Safety	Clinical trial	Status
IDO-1	Epacadostat (INCB24360)	–	Phase 1	Rectal Cancer	–	–	–	–	NCT03516708	Recruiting
		MEDI4736	Phase 1 Phase 2	Solid Tumors Head and Neck Cancer Lung Cancer UC	In the study, attendants were divided into 6 groups, ORR was 16.7%, 0.0%, 25.0%, 0.0%, 12.5%, 22.2% for each group; ORR 12.2% for attendants received Epacadostat (100 mg) + Durvalumab (10 mg/kg), and that was 12.9% for participants received Epacadostat (300 mg) + Durvalumab (10 mg/kg)*	2.4, 12.0, 1.9, 1.7, 4.12.5 1.9 and 2.1	–	Serious adverse events were 2/6, 0/3, 2/4, 0/4, 4/8, 3/9 for each group. Serious adverse events were 20/49, 54/93 for each group	NCT02318277 (ECHO-203)	Completed
		Pembrolizumab	Phase 3	UC	Experimental group (*n* = 44): ORR 31.8%; positive control group (*n* = 49): ORR 24.5%*	–	–	Serious adverse events were 23/43 against 23/49	NCT03361865 (KEYNOTE-672/ECHO-307)	Completed
		Pembrolizumab	Phase 1 Phase 2	Advanced melanoma	–	–	–	–	NCT02178722 (ECHO-202/KEYNOTE-037)	Completed
		Fludarabine Cyclophosphamide	Phase 1	Ovarian Cancer Fallopian Tube Carcinoma Primary Peritoneal Carcinoma	–	–	–	–	NCT02118285	Completed
		Pembrolizumab	Phase 2	Lung Cancer	Experimental group (*n* = 77): ORR 32.5%; control group (*n* = 77): ORR 39.0%*	Experimental group: 6.7; control group: 6.2	NA	Serious adverse events were 23/75 for experimental group, and 29/77 for control group	NCT03322540 (KEYNOTE-654–05/ECHO-305–05)	Completed
		Pembrolizumab	Phase 2	Thymic Carcinoma Thymus Neoplasms Thymus Cancer	1/40 attendant had a complete response, 8/40 participants had partial response, 21/40 participants received stable disease, 10/40 participants suffered progression*	4.2	24.9	2/40 patients suffered myocarditis, one case for hyperglycemia, hepatitis, bullous pemphigoid, and polymyositis each	NCT02364076	Unknown
		Pembrolizumab Platinum-based chemotherapy	Phase 2	Lung Cancer	Experimental group (*n* = 91): ORR 26.4%; control group (*n* = 87): ORR 44.8%*	Experimental group: 8.0; control group: 8.2	NA	Serious adverse events were 47/90 against 41/86	NCT03322566 (KEYNOTE-715–06/ECHO-306–06)	Completed
		Pembrolizumab	Phase 3	UC	Experimental group (*n* = 42): ORR 21.4%; control group (*n* = 42): ORR 9.5%*	–	–	Serious adverse events were 22/42 for experimental group, and 16/41 for control group	NCT03374488 (KEYNOTE-698/ECHO-303)	Completed
		–	Phase 1	Solid Tumors and Hematologic Malignancy	–	–	–	–	NCT01195311	Completed
		Pembrolizumab	Phase 2	Gastrointestinal Stromal Tumors	–	–	–	–	NCT03291054	Completed
		Pembrolizumab Cetuximab Cisplatin Carboplatin 5-Fluorouracil	Phase 3	Head and Neck Cancer	Experimental group (*n* = 35): ORR 31.4%; negative control group (*n* = 19): ORR 21.1%; positive control group (*n* = 35): ORR 34.3%*		–	Serious adverse events were 12/34 for experimental group, 8/19 for negative control group, and 12/34 for positive control group	NCT03358472 (KEYNOTE-669/ECHO-304)	Active, not recruiting
		Low dose cyclophosphamide	Phase 1 Phase 2	Breast Cancer Female Breast Neoplasm Female	–	–	–	–	NCT03328026	Recruiting
	BMS-986205	Nivolumab	Phase 2	Endometrial Adenocarcinoma and Endometrial Carcinosarcoma	–	–	–	–	NCT04106414	Active, not recruiting
		Nivolumab	Phase 3	Melanoma Skin Cancer	–	–	–	Serious adverse events were 4/10 for experimental group against 4/10 for control group*	NCT03329846	Completed
		Nivolumab	Phase 1 Phase 2	Advanced Cancer	–	–	–	Two cases for malignant neoplasm progression, one case for tuberculosis, acute kidney injury, pneumonitis each	NCT03792750	Completed
		–	Phase 1	Cancer	–	–	–	–	NCT03247283	Completed
		Nivolumab Gemcitabine Cisplatin Placebo	Phase 3	Bladder Cancer Muscle-Invasive Bladder Cancer	–	–	–	–	NCT03661320	Recruiting
		Itraconazole Rifampin	Phase 1	Malignancies Multiple	–	–	–	–	NCT03346837	Completed
		Nivolumab	Phase 1	Advanced Cancer	–	–	–	–	NCT03192943	Completed
		Nivolumab Ipilimumab	Phase 1 Phase 2	Advanced Cancer Melanoma NSCLC	–	–	–	–	NCT02658890	Recruiting
CD27	Varlilumab (CDX-1127)	–	Phase 1 Phase 2	B Cell Lymphoma	–	–	–	–	NCT03307746	Active, not recruiting
		nivolumab	Phase 1 Phase 2	HNSCC Ovarian Carcinoma-Enrollment Completed CRC-Enrollment Completed RCC (Phase ll Only) GBM (Phase ll Only)-Enrollment Completed	–	–	–	–	NCT02335918	Completed
		–	Phase 1	Selected refractory or relapsed hematologic malignancies or solid tumors	A patient with mRCC underwent a partial response (78% shrinkage) and had a durable response with PFS > 2.3 years without additional treatment. Eight patients underwent SD > 3 months, including a patient with metastatic RCC with PFS of > 3.9 years	–	–	Only one case with grade 3 transient asymptomatic hyponatremia was reported. Other adverse events were limited to grade 1 or 2 in severity*	NCT01460134	Completed
		Atezolizumab Cobimetinib	Phase 2	Unresectable Liver and Intrahepatic Bile Duct Carcinoma	–	–	–	–	NCT04941287	Recruiting
		ONT-10	Phase 1	Advanced Breast Carcinoma Advanced Ovarian Carcinoma	–	–	–	–	NCT02270372	Completed
		Rituximab	Phase 2	Relapsed or refractory B cell malignancies	–	–	–	Three Grade 3 treatment-related events in the study, including hyponatremia, decreased appetite, and decreased lymphocyte count	ISRCTN15025004	Ongoing
		Atezolizumab	Phase 1	Refractory NSCLC Stage IV Lung Cancer AJCC v8	–	–	–	–	NCT04081688	Recruiting
		IMA950 poly-ICLC	Phase 1	Glioma Malignant Glioma Astrocytoma, Grade II Oligodendroglioma Glioma, Astrocytic Oligoastrocytoma, Mixed	–	–	–	–	NCT02924038	Active, not recruiting
		MHP Montanide ISA-51 poly-ICLC	Phase 1 Phase 2	Melanoma	–	–	–	–	NCT03617328	Recruiting

*Primary endpoint; NSCLC, non-small-cell lung cancer; SCLC, small-cell lung cancer; HNSCC, head and neck squamous cell carcinoma; MSS, microsatellite stable; UC, urothelial cancer; TNBC, triple-negative breast cancer; DLBCL, diffuse large B cell lymphoma; MSI, microsatellite instability

So far, studies on IDO-1 have been extensive, and many drugs have reached phase III of clinical trials. However, there is the unexpected failure of Epacadostat (INCB024360). Epacadostat is a potent and highly selective IDO-1 enzyme inhibitor that decreases tryptophan metabolism, resulting in the enhanced viability of effector T cells, NK cells, CD86^high^ DCs, decreased apoptosis, and the reduced expansion of Tregs [114].

- In phase I and II studies, for example, ECHO-202/KEYNOTE-037 (NCT02178722), the result indicated excellent tolerance and efficacy of combination therapy, including Epacadostat plus Pembrolizumab that targets advanced melanoma. However, in a phase III study, the combination of Epacadostat and Pembrolizumab showed no superiority over Pembrolizumab monotherapy. Hence, scientists try to resolve this by exploring the efficacy of Epacadostat in other solid tumors. In KEYNOTE-672/ECHO-307 (NCT03361865), another phase III study, Epacadostat, along with Pembrolizumab, was applied to patients with urothelial cancer (UC). Participants were given Pembrolizumab 200 mg intravenously, while one group (44 participants) received Epacadostat 100 mg BID orally twice daily, and the other (49 participants) took a placebo instead. The result showed that patients in the Pembrolizumab + Epacadostat group had an ORR of 31.8%, compared to 24.5% in the Pembrolizumab + Placebo group. Twenty-three out of 43 patients and 23 out of 49 reported severe adverse events. It is promising news that Epacadostat did improve the therapy, but further studies are needed to explore its effect on tolerance.
- A phase II clinical trial about the combined therapy of Epacadostat plus Pembrolizumab in patients suffering advanced solid tumors showed an encouraging anti-tumor response (NCT03322540).

Recently, a series of researches were restarted to increase the efficacy of Epacadostat by combining Epacadostat with other drugs, vaccines, and radiation in glioblastoma (NCT03532295), metastatic pancreatic adenocarcinoma (NCT03006302), or breast cancer in females (NCT03328026), but no results have been reported yet.

Besides Epacadostat, other antibodies targeting IDO were developed to explore IDO target therapy in solid tumors. The study of PF-06840003 that was developed by Pfizer (NCT02764151) was terminated, and anti-IDO-1 agent LY3381916 was also abandoned. Encouraging news came from BMS-986205, another IDO-1 inhibitor.

- A study (NCT03792750) concerning advanced tumors in Chinese where BMS-986205 was combined with Nivolumab. Twelve participants were enrolled, 11 experienced adverse events, 3 underwent severe adverse events, and 5 discontinued due to experiencing adverse events. There were two cases of malignant neoplasm progression and one of tuberculosis, acute kidney injury, and pneumonitis.
- In one study (NCT03329846), researchers enrolled 20 patients suffering from melanoma and skin cancer to investigate the safety of combination therapy of BMS-986205 with Nivolumab compared to Nivolumab only. The result showed that four patients underwent severe adverse events in both groups. The study has been completed, and the outcome indicated that BMS-986205 has little effect on tolerance. However, this phase III study intended to enroll 700 participants and ended up reporting only 20 cases, and there were no other results reported either. These indicated a relatively unsuccessful study, and a more extensive sample study is needed to add to the stringency.

Among all studies concerning IDO-1, Epacadostat is of great potential. Though its trial on melanoma was not satisfying, its efficacy for urothelial cancer was promising.

## CD27

### Structure and function

**Fig. 2:**
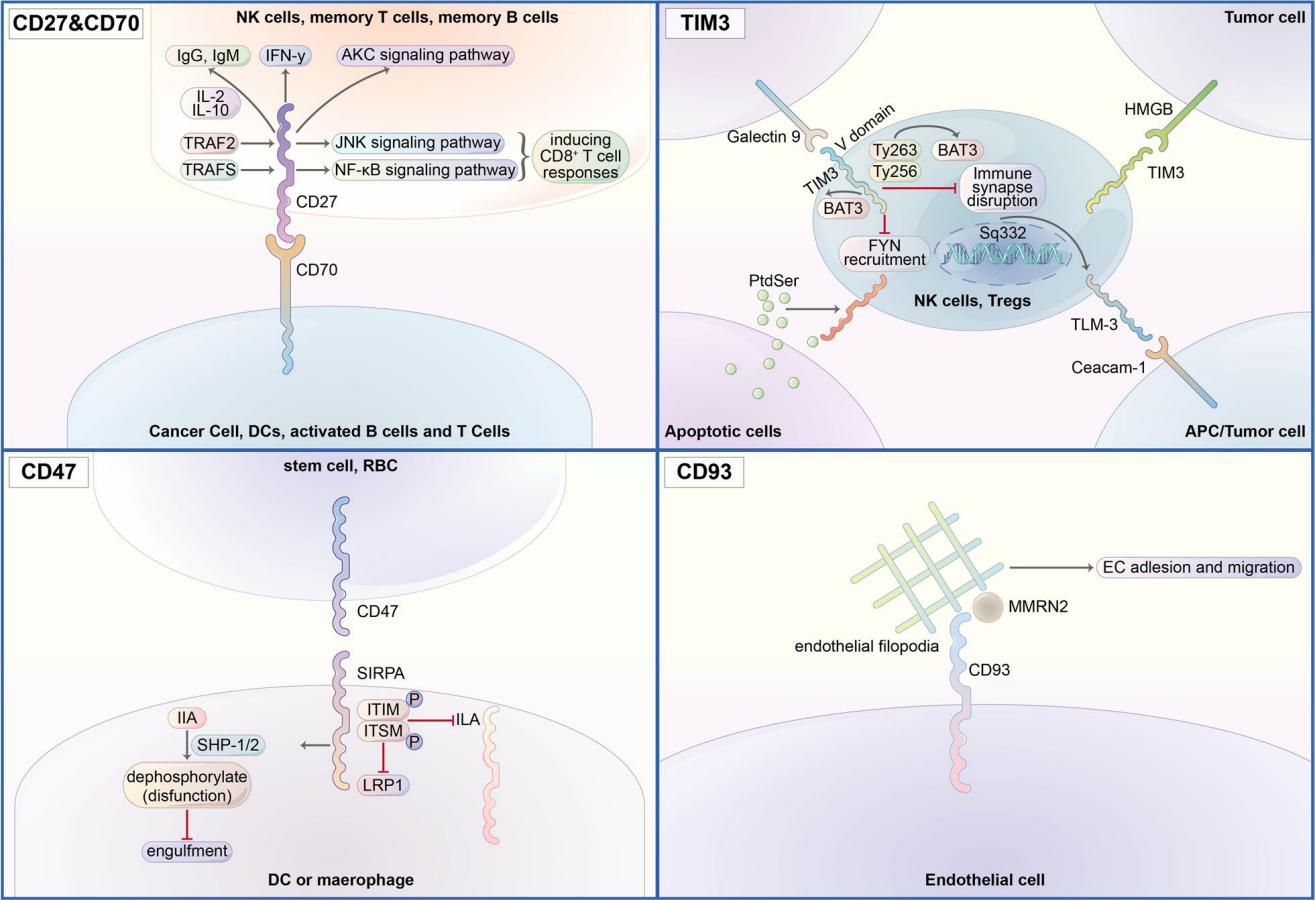
Mode of action of CD27/CD70, TIM-3, CD47, and CD93 signaling pathways

CD27 is a member of the TNF receptor superfamily (TNFRSF). Besides, it is a type I transmembrane, disulfide-linked homodimer [115] (Fig. 2). CD27 is widely expressed in human lymphocytes, including naive and central memory T (TCM) cells, germinal center and memory B cells, plasma cells, and NK cells [116]. As the unique ligand of CD27, CD70 is mainly expressed in hematologic malignancies, for example, diffuse large B cell, follicular lymphoma, Hodgkin lymphoma, Waldenström macroglobulinemia, multiple myeloma, human T-lymphotropic virus type 1- and EBV-associated malignancies [116]. The costimulatory signals induced by CD70-CD27 interaction increase T cell proliferation and activity [117]. Unlike CD27, CD70 expression is transitory and limited to a subset of strongly activated T cells, B cells, and DCs, but is undetectable in homeostasis [118]. And CD70 expression is also being detected in nonhematologic malignancies, for example, renal cell carcinoma and glioblastoma [119].

After binding to CD70, the TRAF2 and TRAF5 adaptor proteins are recruited to the cytoplasmic tail of CD27, which activates either the JNK or NF-kb signaling pathways [115, 120] and finally induces the effective primary and memory cell-mediated responses. There was evidence that anti-CD70 treatment could alleviate Th17-cell-mediated inflammatory disease and experimental autoimmune encephalomyelitis (EAE) on CD4+ T cells. Besides, CD70-transgenic mice were detected with increased interferon-γ-producing CD4+ and CD8+ T cells [117].

Apart from the typical costimulatory pathway, other mechanisms account for the CD27 expression on B cells and NK cells. In human peripheral B cells cultivated with IL-2 and IL-10, the binding of CD27 with CD70 upregulates the secretion of IgG and IgM. Plasmacytoid dendritic cells (pDCs) are believed to be another important source of CD70 [121] that plays a vital role in regulating the differentiation of B cell [122]. CD27 has also been proved to stimulate Akt signaling in NK cells and upregulate IFN-γ secretion [115].

### Clinical trials on CD27 (Table 4)

So far, the only fully human immunoglobulin G1 kappa anti-CD27 monoclonal antibody [123] is Varlilumab (CDX-1127), which interacts with the CD70-binding site and serves as an agonist of CD27.

- In phase I clinical trial exploring the safety and pharmacokinetics of CDX-1127 in patients suffering from selected refractory or relapsed hematologic malignancies or solid tumors (NCT01460134). Fifty-six patients participated in the study until March 2014. In a 3 + 3 dose escalation design (*n* = 25), the patients received a single dose of Varlilumab (0.1, 0.3, 1.0, 3.0, or 10 mg/kg IV) to determine an applicable dose. Base on the data of dose escalation cohort, expansion cohorts were initiated at 3.0 mg/kg in patients with melanoma (*n* = 16) and RCC (*n* = 15). After only one cycle of Varlilumab treatment, a patient with metastatic RCC underwent a partial response (78% shrinkage) and had a durable response with PFS > 2.3 years without additional treatment. Eight patients underwent SD > 3 months, including a patient with metastatic RCC with PFS of > 3.9 years. Only one case with grade 3 transient asymptomatic hyponatremia was reported. Other adverse events were limited to grade 1 or 2 in severity. The study confirmed that at 10 mg/kg, Varlilumab showed a good tolerance, while the maximum tolerated dose remains unknown, and Varlilumab has clinical activity. Since two RCC patients had extremely long PFS, and the recruitment of 90 has been completed, we expect the ITT group's analysis and further analysis, which show more favorable results for patients with RCC and other mechanisms [124].
- In another phase II study, researchers focused on the cooperation of Rituximab and Varlilumab while applying to relapsed or refractory B cell malignancies (ISRCTN15025004), which demonstrated depleted B cells and increased infiltration of myeloid cells [125]. Forty patients suffering low- or high-grade relapsed or refractory CD20+ B cell lymphoma were enrolled. Participants were divided into two groups, receiving different doses of Varlilumab; the result has not come out yet, but researchers reported three Grade 3 treatment-related events in the study, including hyponatremia, decreased appetite, and decreased lymphocyte count [125].
- Another study exploring the effect of Varlilumab and Nivolumab in advanced refractory solid tumors (NCT02335918) is completed nowadays, and the result is about to publicly available.

## CD70

CD70 is a member of the TNF ligand superfamily. It is mainly expressed on activated T cells, B cells, and DCs [126]. By binding to CD27, its specific ligand, it sends costimulatory signals. Then researchers found a significant enhancement in T cell activation, survival, proliferation, and differentiation [121, 126]. By the way, ligation of CD70 on NK cells upregulated Akt signaling, which is well known for activating NK cells’ pathway [127]. It is undisputed that CD70 and CD27 weigh a lot in immunity. At the same time, many terrible cases have also witnessed the CD27–CD70 axis's role in many inflammatory settings [126, 128]. For example, in MRL/lpr lupus-prone mice, the study showed defective DNA methylation and CD70 overexpression [129]. On the contrary, if some biallelic mutations are found on the CD27-encoded gene or the gene encoding the CD27’s ligand, CD70. Inborn errors of immunity will occur, and it can finally bring a list of EBV (Epstein–Barr virus)-associated immunopathologic conditions like lymphoproliferative disorders (LPDs) or hemophagocytic lymphohistiocytosis (HLH) [116, 130]. Similar things happened in tumor cells, in which an NLRandP3-mediated release of IL-18 would downregulate CD70 on tumor cells and generate immune escape [131].

However, recent studies reported overexpression of CD70 on multiple tumor cells, like solid cancers, for example, renal cell cancer (RCC), glioblastoma, and hematological malignancies [132–134]. Advanced research showed, at least in clear cell renal cell carcinoma (ccRCC), CD70 upregulation is driven by HIF, which ought to be hydrolyzed by pVHL (VHL protein), and the story started with Von Hippel–Lindau gene (VHL) mutations in ccRCC [135]. A further rigid mechanism of overexpression of CD70 on tumors remains unknown, but CD70 is still attractive as a therapeutic target.

### Clinical trials on CD70 (Table 5)

**Table 5:** Clinical trials on TIM-3, BTLA, CD47, B7-H3, B7-H4, CD70, and TIGIT

Target	Drug	Combination agent	Phase	Tumor type	Clinical efficacy	PFS	OS	Safety	Clinical trial	Status
TIM-3	Sym023	–	Phase 1	Metastatic Cancer Solid Tumor Lymphoma	3 patients were reported SD > 16 weeks, 7 were reported SD ≤ 16 weeks. ORR not reported	–	–	Researchers reported one case of immune-mediated arthritis for Sym023 0.1 mg/kg, one case of lipase increased and pathological fracture each for 10.0 mg/kg, and one case of back pain plus spinal cord compression for 20.0 mg/kg*	NCT03489343	Completed
		Sym021 Sym022	Phase 1	Metastatic Cancer Solid Tumor	–	–	–	–	NCT04641871	Recruiting
		Sym021 Sym022	Phase 1	Metastatic Cancer Solid Tumor Lymphoma	–	–	–	–	NCT03311412	Completed
	MBG453 (Sabatolimab)	Venetoclax Azacitidine	Phase 2	Acute Myeloid Leukemia	–	–	–	–	NCT04150029	Recruiting
		Azacitidine	Phase 3	MDS Leukemia Myelomonocytic Chronic	–	–	–	–	NCT04266301	Active, not recruiting
		Hypomethylating agents	Phase 2	MDS	–	–	–	–	NCT03946670	Active, not recruiting
		HDM201 Venetoclax	Phase 1	AML High-risk MDS	–	–	–	–	NCT03940352	Recruiting
		PDR001 Decitabine	Phase 1 Phase 2	Advanced Malignancies	ORR in the monotherapy group was 0% and DCR was 29% with 25/87 SD* ORR in the combination group was 5% and DCR was 44% with 4/86 PR and 34/86 SD*	–	–	One DLT in combination cohort (grade 4 MG)* 11% developed grade 3 or 4 AEs in the combination cohort*	NCT02608268	Active, not recruiting
		–	Phase 1	GBM	–	–	–	–	NCT03961971	Recruiting
		Azacitidine Decitabine INQOVI (oral decitabine)	Phase 2	MDS	–	–	–	–	NCT04878432	Recruiting
		Sabatolimab Azacitidine	Phase 1 Phase 2	Acute Myeloid Leukemia	–	–	–	–	NCT04623216	Recruiting
		NIS793 canakinumab	Phase 1	MDS	–	–	–	–	NCT04810611	Recruiting
		Sabatolimab, azacitidine, venetoclax	Phase 2	MDS	–	–	–	–	NCT04812548	Recruiting
	TSR-022	TSR-042	Phase 2	Adult Primary Liver Cancer Advanced Adult Primary Liver Cancer Localized Unresectable Adult Primary Liver Cancer	–	–	–	–	NCT03680508	Recruiting
		TSR-042	Phase 2	Melanoma Stage III Melanoma Stage IV	–	–	–	–	NCT04139902	Recruiting
		–	Phase1	Advanced solid tumors	–	–	–	–	NCT02817633	Recruiting
	RO7121661	–	Phase 1	Solid Tumors Metastatic Melanoma NSCLC SCLC ESCC	–	–	–	–	NCT03708328	Active, not recruiting
		RO7247669 Nivolumab	Phase 2	Advanced or Metastatic ESCC	–	–	–	–	NCT04785820	Recruiting
	LY3321367	LY3300054	Phase 1	Solid Tumor	In monotherapy expansion cohort, outcomes varied: anti-PD-1/L1 refractory patients [*N* = 23, ORR 0%, DCR 35%, PFS 1.9 months] versus anti-PD-1/L1 responders (*N* = 14, ORR 7%, DCR 50%, PFS 7.3 months). In combination expansion cohorts (*N* = 91), ORR and DCR were 4% and 42%	1.9, 7.3(detailed in Clinical efficacy)	–	No DLTs were observed*, treatment-related adverse events (≥ 2 patients) included pruritus, rash, fatigue, anorexia, and infusion-related reactions	NCT03099109	Active, not recruiting
		LY3300054 Ramucirumab Abemaciclib Merestinib	Phase 1	Solid Tumor MSI-H Solid Tumors Cutaneous Melanoma Pancreatic Cancer Breast Cancer (HR + HER2-)	–	–	–	DLTs associated with hepatotoxicity were observed in 3 of 4 patients in the abemaciclib lead-in cohorts. No DLTs or grade 3 or 4 hepatotoxicity were reported in the concurrent abemaciclib arm*	NCT02791334	Active, not recruiting
BTLA	JS004	–	Phase 1	Advanced Solid Tumors	–	–	–	–	NCT04773951	Recruiting
		Toripalimab	Phase 1 Phase 2	Advanced Lung Cancer	–	–	–	–	NCT05000684	Recruiting
		–	Phase 1 Phase 2	HNSCC Nasopharyngeal Carcinoma	–	–	–	–	NCT04929080	Recruiting
		–	Phase 1	Recurrent/Refractory Malignant Lymphoma	–	–	–	–	NCT04477772	Recruiting
		–	Phase 1	Advanced Solid Tumor	–	–	–	–	NCT04278859	Unknown
		Toripalimab	Phase 1	Advanced Unresectable Solid Tumor Metastatic Solid Tumor	–	–	–	–	NCT04137900	Recruiting
		Toripalimab	Phase 1	Advanced Unresectable Solid Tumor Metastatic Solid Tumor	–	–	–	–	NCT04137900	Recruiting
CD47	Magrolimab (Hu5F9-G4)		Phase 1	Solid tumors	ORR ~ 5% DCR 19% with 2/43 PR (ovarian and fallopian tube cancers) and 6/43 SD (CRC)*	–	–	AEs occurred with higher doses. These included constitutional symptoms (50%), headache (34%), anemia (39%), and lymphopenia (28%) *	NCT02216409	Completed
		Rituximab Gemcitabine Oxaliplatin	Phase 1 Phase 2	Non-Hodgkin Lymphoma	CRR 21% ORR 49% with 16/75 CR and 21/75 PR	–	–	DLTs 4% (no specifics provided) *	NCT02953509	Active, not recruiting
		Venetoclax Azacitidine Cytarabine Daunorubicin Idarubicin Steroidal Eye Drops	Phase 3	AML	–	–	–	–	NCT04778397	Recruiting
	AK117	–	Phase 1	Neoplasms Malignant	–	–	–	–	NCT04728334	Recruiting
		Azacitidine	Phase 1 Phase 2	Myelodysplastic Syndrome	–	–	–	–	NCT04900350	Recruiting
		Azacitidine	Phase 1 Phase 2	Acute Myeloid Leukemia	–	–	–	–	NCT04980885	Recruiting
			Phase 1	Neoplasms Malignant	–	–	–	–	NCT04349969	Not yet recruiting
		AK112 Nab-paclitaxel paclitaxel	Phase 2	Metastatic Triple-negative Breast Cancer Locally Advanced Triple-negative Breast Cancer	–	–	–	–	NCT05227664	Recruiting
		Capecitabine tablets Oxaliplatin Cisplatin Paclitaxel Irinotecan Docetaxel 5-FU AK104	Phase 1 Phase 2	Advanced Malignant Tumors	–	–	–	–	NCT05235542	Not yet recruiting
	TTI-622	–	Phase 1	Relapsed or refractory lymphomas	1 patient (DLBCL) with 5 prior lines of therapy achieved a PR by week 8 and a CR by week 36	–	–	No DLTs*	NCT03530683 (TTI-622–01)	Recruiting
	RRx-001	Nivolumab	Phase 1	Advanced solid malignancies or lymphomas	ORR 25%, DCR 67% with 3/12 PR, 5/12 SD, and 3/12 PD	–	–	No DLTs reported 1 patient discontinued therapy due to pneumonitis*	NCT02518958 (PRIMETIME)	Completed
B7-H3	MGC018	MGA012	Phase 1 Phase 2	Advanced Solid Tumor, Adult Metastatic Castrate Resistant Prostate Cancer NSCLC TNBC HNSCC Melanoma Prostate Cancer	ORR 0% and DCR 15% with 3/20 SD	–	–	1 DLT: grade 4 neutropenia 3 serious AEs: pneumonitis, gastroenteritis, stasis dermatitis*	NCT03729596	Recruiting
	B7-H3 CAR-T	Temozolomide	Phase 1	Recurrent GBM Refractory GBM	–	–	–	–	NCT04385173	Unknown
		Temozolomide	Phase 1 Phase 2	Recurrent GBM Refractory GBM	–	–	–	–	NCT04077866	Recruiting
		–	Phase 1	Lung Cancer Immunotherapy CAR-T Cell	–	–	–	–	NCT03198052	Recruiting
		Fludarabine Cyclophosphamide	Phase 1	Epithelial Ovarian Cancer	–	–	–	–	NCT04670068	Recruiting
		–	Early Phase 1	Osteosarcoma Neuroblastoma Gastric Cancer Lung Cancer	–	–	–	–	NCT04864821	Not yet recruiting
		–	Phase 1 Phase 2	Solid Tumor	–	–	–	–	NCT04432649	Recruiting
		GD2, PSMA CAR-T cells	Phase 1 Phase 2	Neuroblastoma	–	–	–	–	NCT04637503	Recruiting
	Enoblituzumab (MGA271)	–	Phase 1	Neuroblastoma Rhabdomyosarcoma Osteosarcoma Ewing Sarcoma Wilms Tumor Desmoplastic Small Round Cell Tumor	–	–	–	–	NCT02982941	Completed
		Ipilimumab	Phase 1	Melanoma NSCLC	–	–	–	–	NCT02381314	Completed
		–	Phase 2	Prostate Cancer	31% of patients had a more than 10% decline in PSA before post-prostatectomy, and an altered Gleason score was observed*	–	–	3 cases of serious adverse events*, including ascites, pericardial effusion, cardiac disorders, atrial fibrillation, pericarditis, myocarditis, and infusion-related reaction	NCT02923180	Active, not recruiting
		Retifanlimab Tebotelimab	Phase 2	Head and Neck Cancer Head and Neck Neoplasms HNSCC	–	–	–	–	NCT04634825	Recruiting
		IP FT516 IL-2	Phase 1	Ovarian Cancer Fallopian Tube Adenocarcinoma Primary Peritoneal Cavity Cancer	–	–	–	–	NCT04630769	Completed
		Pembrolizumab MGA012	Phase 1	Melanoma Head and Neck Cancer NSCLC UC	ORR 33.3% for patients with CPI-naïve HNSCC, and 35.7% for patients with CPI-naïve NSCLC	–	–	Treatment-related adverse events occurred in 116 patients (87.2%) and were grade ≥ 3 in 28.6%*	NCT02475213	Completed
B7-H4	FPA150	–	Phase 1	B7-H4 positive solid malignancies	ORR 3% and DCR 38% with 1/29 PR and 10/29 SD	–	–	No DLTs or grade 4/5 toxicities were reported	NCT03514121	Completed
CD70	SGN-CD70A	–	Phase 1	CD70-positive metastatic RCC	One patient in the 50-μg/kg cohort achieved a PR (6%), and 13 out of 18 patients (72%) had SD. Thus, the overall disease control rate was 78%, and the estimated median PFS was 3.5 months	3.5 months	–	Grade 3 TEAEs were thrombocytopenia (22%), anemia (17%), neutropenia (17%), and dehydration (11%)*	NCT02216890	Completed
	Cusatuzumab	Azacitidine and Venetoclax	Phase 1	AML	–	–	–	–	NCT04150887	Active, not recruiting
TIGIT	Tiragolumab	Atezolizumab	Phase 2	NSCLC	ORR 31.3% in experimental group vs 16.4% in the control group*	5.4 months vs 3.6 months*	–	14 (21%) patients receiving combination therapy and 12 (18%) patients receiving monotherapy had serious adverse effects, two treatment-related deaths	NCT03563716	Active, not recruiting
		Atezolizumab	Phase 3	NSCLC	–	–	–	–	NCT04294810 (SKYSCRAPER-01)	Recruiting
		Atezolizumab, Carboplatin and Etoposide	Phase 3	ES-SCLC	–	5.4 months in the experimental group vs 5.6 months in the control group*	Both 13.6 months*	Grade 3/4 TRAEs occurred in 52.3% of the experimental group and 55.7% of the placebo group and Grade 5 TRAEs occurred in 0.4% of experimental group and 2.0% placebo group	NCT04256421 (SKYSCRAPER-02)	Active, not recruiting
	Etigilimab (OMP-313M32)	Nivolumab	Phase 1	Advanced or metastatic cancer	No DLT detected. The maximum administered dose was 20 mg/kg. Besides, in the phase Ia study, seven patients (30.0%) had SD, and no PR was reported; in the phase Ib study, one patient had a partial response. One patient had prolonged SD of nearly eight months	56.0 days in phase 1a, 57.5 days in phase 1b	–	The most reported AEs were rash (43.5%), nausea (34.8%), and fatigue (30.4%) in phase Ia and decreased appetite (50.0%), nausea (50.0%), and rash (40%) in Phase Ib. Six patients experienced Grade ≥ 3 treatment-related AEs*	NCT03119428	Terminated

*Primary endpoint; NSCLC, non-small-cell lung cancer; TNBC, triple-negative breast cancer; MSS, microsatellite stable; HNSCC, head and neck squamous cell carcinoma; ESCC, esophageal squamous cell carcinoma; SCLC, small-cell lung cancer; MDS, myelodysplastic syndrome; AML, acute myeloid leukemia; CRC, colorectal cancer; RCC, renal cell carcinoma; GBM, glioblastoma; UC, urothelial cancer

At present, clinic trials on CD70 mainly focus on ADC (antibody–drug conjugate), mAb (monoclonal antibody), and CAR (chimeric antigen receptor)-T therapy. Though its overexpression fails to rouse immune response, CD70 can still be used to indicate tumor cells.

- SGN-CD70A is a CD70-targeted antibody–drug conjugate. In a phase I trial on participants suffering CD70-positive, metastatic RCC (NCT02216890), 18 patients were enrolled, 94% had the clear cell subtype of RCC, and all participants received SGN-CD70A IV in dose escalation (8, 15, 30, 50, 80, 120, 160, and 200 μg/kg). One patient in the 50-μg/kg cohort achieved a PR (6%), and 13 out of 18 patients (72%) had SD. Thus, the overall disease control rate was 78%, and the estimated median PFS was 3.5 months [136]. Grade 3 TEAEs were thrombocytopenia (22%), anemia (17%), neutropenia (17%), and dehydration (11%), and there were no reports of neutropenic fever. The result did not support its development in mRCC, but there is still a chance in combination therapy.

Besides ADC, mAb Cusatuzumab was developed by Argenx, and studies showed that it could both induce cytotoxicity against CD70+ tumor cells through enhanced ADCC, complement‐dependent cytotoxicity, or Ab‐dependent cellular phagocytosis) and improve the anti-tumor immune response by interrupting the CD70‐CD27 signaling with Tregs [137]. In recent studies, researchers reported that combination therapy of Cusatuzumab plus Azacitidine has a higher ORR than monotherapy of Azacitidine in patients with phase I or 2 AML.

- In a phase I study, Cusatuzumab was applied with Azacitidine and Venetoclax to patients suffering AML (NCT04150887). Sixty-one participants were divided into two cohorts, receiving combination therapy of Cusatuzumab + Venetoclax, or Cusatuzumab + Venetoclax + Azacitidine. The study is not recruiting now, hoping it will bring us a satisfying outcome.

The third CD70-related treatment is CAR-T, which consists of diverse T‐lymphocytes and CAR transmembrane molecules encoded by artificial fusion genes. It redirects T cell cytotoxicity against cells expressing a specific target antigen. CAR binding to target antigens is independent of the MHC receptor, resulting in abundant T cell activation and robust anti-tumor responses [138]. Therefore, CAR-T therapy was born for tumor immunotherapy. In one study, researchers constructed seven anti-human CD70 CARs. They cured NSG mice bearing established human tumors that secreted CD70 and human lymphocytes transduced with the CAR, which side effect includes transient weight loss and hematopoietic suppression [139]. A similar trial reported favorable outcomes that CD70-specific CAR-T cells could recognize and kill CD70-positive head and neck squamous cell carcinoma cells efficiently [140]. ALLO-316 is an allogeneic CAR-T cell therapy targeting CD70, and the phase I study has not been completed yet.

Research on ADCs or mAbs of CD70 is relatively few, but it has nothing to do with its efficacy on AML, especially in combination with Azacitidine. CAR-T therapy is vital in immune therapies targeting CD70, and further studies are promising.

## TIM-3

### Structure and function

T cell immunoglobulin and mucin domain-containing protein 3 is a member of the TIM family of immunoregulatory proteins [50]. TIM-3 was initially classified as a receptor expressed on IFN-γ-producing CD4+ Th1 and CD8+ T cytotoxic 1 (Tc1) T cells [101]. Recent studies have also shown its capacity for immune evasions like PD-1 and CTLA-4. TIM-3 is encoded by HAVCR2 and located on chromosome band 5q33.2 in humans. Human TIM-3 protein comprises an amino-terminal immunoglobulin variable domain (V domain) with five noncanonical cysteines, a mucin stalk, a transmembrane domain, and a cytoplasmic tail. Unlike other immune checkpoint molecules, for example, PD-1 and TIGIT. The TIM-3 cytoplasmic tail has no classical inhibitory signaling motifs, like immune receptor tyrosine-based inhibitory motif (ITIM) or immune receptor tyrosine-based switch motif (ITSM). Instead, TIM-3 obtains five conserved tyrosines. Among the five tyrosines, Tyr256 and Tyr263 allow interactions with HLA-B-associated transcript 3 (BAT3) and the tyrosine kinase FYN. TIM-3 was firstly found on IFN-γ-producing CD4+ and CD8+ T cells. Gradually, Tregs, myeloid cells, NK cells, and mast cells are also revealed to express TIM-3 abundantly [141, 142].

TIM-3 is also reported with four distinct ligands, including galectin-9, HMGB1, carcinoembryonic antigen cell adhesion molecule 1 (Ceacam-1), and phosphatidyl serine (PtdSer) [50]. Galectin-9 is a C-type lectin widely expressed and secreted by many hematopoietic cells. Galectin-9 binds to carbohydrate moieties on cell surface proteins, which induces intracellular calcium influx and cell death of TIM-3+ T cells. The discovery of PtdSer could be contributed to elucidating the crystal structures of the TIM family. PtdSer binds to the pocket framed by the FG and CC′ loops in the TIM-3 immunoglobulin V domain [143], acts as a surface marker for apoptotic cells, and coordinates calcium-binding. The binding site of TIM-3 and HMGB1 remains uncertain, and their interaction was proposed to impact innate immune activation. Ceacam-1 works in the regulation of antiviral responses [102].

### Clinical trials on TIM-3 (Table 5)

There are over ten TIM-3 antagonistic mAbs being registered on ClinicalTrials.gov. Sym023 is a recombinant anti-TIM-3 monoclonal human antibody.

- The first study targeting Sym023 was started in 2018 and was intended to investigate the safety and antineoplastic activity of Sym023 on patients suffering from advanced solid tumors or lymphomas (NCT03489343). Twenty-four patients were enrolled and distributed into six groups, each corresponding to a dose level. Two cases (66.7%) in the group with Sym023 1.0 mg/kg and Sym023 3.0 mg/kg each reached SD ≤ 16 weeks. One case in the 0.1 mg/kg group reached SD > 16 weeks. For patients in the 20.0 mg/kg group, there were 83.3% reached SD, but no DLTs were reported. It also reported severe adverse events, including immune-mediated arthritis (1/1), pathological fracture (1/7), back pain (1/6), and spinal cord compression (1/6). In the subsequent trial, researchers could try larger doses of Sym023. The success of Sym023 promoted the studies evaluating the preliminary efficacy of the combined Sym021 (anti-PD-1), Sym022 (anti-LAG-3), and Sym023 in tumor therapies (NCT04641871 and NCT03311412).
- Novartis also reported the success of anti-TIM-3 antibody MGB453 (Sabatolimab) as a single agent or cooperated with PDR001 in phase I clinical trial in patients suffering advanced malignancies (NCT02608268), in which 219 participants were given MBG453 monotherapy (*n* = 133) or combination therapy of MBG453 plus PDR001 (*n* = 86). The result showed that the RP2D for Novartis was selected as 800 mg Q4W. The most common adverse event in the study was treatment-related fatigue (9%, Novartis; 15%, combination). One out of 151 patients in the dose-determining set underwent a DLT: grade 4 myasthenia gravis. Overall, 111 patients (51%) experienced grade 3/4 events. Further studies are warranted to identify how much patients will benefit from MBG453 and PDR001 therapy.
- Other TIM-3 inhibitors like INCAGN2390, LY3321367, BMS-986258, and SHR1702 are also being tested in phase I trial independently (INCAGN02390, NCT03652077) or in combination with anti-PD-1/PD-L1 mAb (LY3321367, NCT03099109. BMS-986258, NCT03446040. SHR1702, NCT03871855) in advanced malignancies.

## CD47

The CD47 protein (also known as integrin-associated protein, IAP) consists of a single extracellular V-set IgSF domain, a presenilin domain with five membrane-spanning sections, and a short cytoplasmic domain that is subject to alternative splicing [144, 145], it is cell surface protein initially observed on stem cells, and soon being found expressed by most cell types including RBCs [146]. The cell surface protein CD47 is a “Don’t Eat Me” signal that protects healthy cells from macrophage engulfment [147], so lacking CD47 would bring hematopoietic cells a rapid engulfment from macrophages and trigger DCs activation [148]. When functioning in the nervous system, CD47 protects active synapses from pruning by microglia [149]. But signal could also be found on cancer cells, thus mediating evading immune detection [150, 151]. On the contrary, anti-CD47 therapies achieved encouraging results [152, 153]. In short, more research is needed to explore the mechanism of CD47 on how it suppresses macrophage engulfment. Using CD47 signaling for tumor therapies is feasible and attractive.

SIRPA (signal regulatory protein α) is the primary receptor of CD47 and is mainly expressed on macrophages or dendritic cells [148, 154, 155] and also in neurons, endothelial cells, and fibroblasts. SIRPA has three extracellular Ig-like domains, one distal IgV-like domain, and two membrane-proximal IgC-like domains [156, 157], and the intracellular region of SIRPα obtains both ITIM and ITSM motifs. The two are critical for the inhibitory activity of the receptor. When binding to CD47, the ITIM or ITSM would be phosphorylated and counteract the cellular activation that occurs as an activating receptor (most likely an ITAM-containing receptor). Meanwhile, tyrosine phosphatases SHP-1 and SHP-2 will be gathered and triggered in [157]. Studies have shown that the two phosphatases will dephosphorylate motor protein myosin IIA of macrophages and dendritic cells, thus inhibiting phagocytosis [158, 159]. However, details on CD47 binding translating across the cell membrane and finally driving SIRPA phosphorylation remain unknown [160].

An exciting phenomenon arose with the wide application of anti-CD47 antibodies: fewer normal cells seem to be affected. In contrast, the blockade of CD47 with a monoclonal antibody enables phagocytosis of tumor cells [150]. Surface calreticulin (CRT) is one of them. It links to its macrophage receptor, low-density lipoprotein-related protein (LRP), and mediates the target cells' engulfment [161]. Researchers found a boom in CRT expression on tumor cells, but the overexpression of CD47 counterbalances the possible phagocytosis it mediated. When anti-CD47 antibodies were used, the overmuch CRT on tumor cells would bind to LRP first, leading to neoplasm preferentially elimination [162].

Besides, it was found that when phagocytes took tumor cells, the cytosolic DNA, especially mtDNA, plays a unique role in anti-tumor immunity. Tumor mtDNA directly interacted with cGAS in the cytosol of DCs, sparking the cGAS-STING-IRF3 signaling pathway and eventually initiating IFN-β production and cross-prime CD8+ T cells [163]. DCs could maintain an alkaline phagosomal lumen by NOX2 (NADPH oxidases II) in the hope of DNA degradation delay, while CD47 could activate SIRPα signaling and downregulate NOX2 [158]. It added to the anti-tumor liveness of anti-CD47 mAbs.

### Clinical trials on CD47 (Table 5)

To date, there are 34 records on CD47 antagonistic mAbs registered on ClinicalTrials.gov. Magrolimab (Hu5F9-G4) is an anti-CD47 antibody and firstly entered the phase I clinical trial in 2014 [164].

- The research (NCT02216409) was intended to verify the safety and tolerability of Magrolimab while applied to solid tumors and made a success. Sixty-two patients were treated: 11 in part A, 14 in B, 22 in C, and 15 in the biopsy cohort. In part A, patients were given doses ranging from 0.1 to 3 mg/kg, and finally, 1 mg/kg was decided to be the priming dose. In later parts, patients were tested for a proper maintenance dose, and the result was a priming dose at 1 mg/kg on the first day and followed by maintenance doses of up to 45 mg/kg weekly showed tolerability with patients. Finally, two patients with ovarian/fallopian tube cancers had partial remissions for 5.2 and 9.2 months. Most participants only underwent mild-to-moderate toxicities (grade 1 or 2), including transient anemia (57%), hemagglutination on a peripheral blood smear (36%), fatigue (64%), etc. [164].
- Advanced studies showed that Magrolimab was applied to patients with non-Hodgkin lymphoma along with Rituximab, Gemcitabine, and Oxaliplatin [165] (NCT02953509). The study enrolled 22 patients, 15 with DLBCL and 7 with follicular lymphoma. All the participants were administered Magrolimab intravenously at a priming dose of 1 mg/kg with weekly maintenance doses of 10 to 30 mg/kg. Researchers reported that the most common toxicity of Magrolimab was the expected on-target anemia and infusion-related reactions, whose intensity was limited to grade 1 or 2. A total of 50% of the patients had an objective response, with 36% having a complete response. The ORR and CR were 40% and 33%, respectively, among patients with DLBCL and 71% and 43%, respectively, among those with follicular lymphoma [165]. More studies are wanted for a more robust outcome. Besides, it is also essential to expand the sample capacity to search for potentially clinically significant safety events.
- Besides lymphoma, a promising study (NCT04778397) compared the efficacy of Magrolimab + Azacitidine against Venetoclax + Azacitidine in adults with AML, but the outcome remains unknown.

AK117 is another anti-CD47 antibody with high expectations, nine clinical trials are registered on Clinicaltrials.gov, but no results are reported.

## CD93

CD93 is a transmembrane receptor found overexpressed in tumor vessels of varied cancer types. CD93 is mainly expressed on endothelial cells and obtains a C-type lectin domain, 5 EGF-like repeats, a serine/threonine-rich mucin-like domain, a transmembrane domain, and a short cytoplasmic domain harboring a binding site for moesin. In endothelial cells, CD93, as a part of the endothelial filopodia, promotes filopodia through a tight binding with Multimerin-2 (MMRN2) [166]. That belongs to the EDEN family and mostly be found in the extracellular environment of tumor vasculature. CD93 is of great importance in EC adhesion and migration that the CD93-MMRN2 complex mediates tumor angiogenesis by forming a fibrillar fibronectin network [167]. Since the abnormal vasculature is a critical pathological feature facilitating tumor outgrowth and metastasis, the blockade of CD93 has been proved to contribute to immunotherapy response [168].

## CD161

**Fig. 3:**
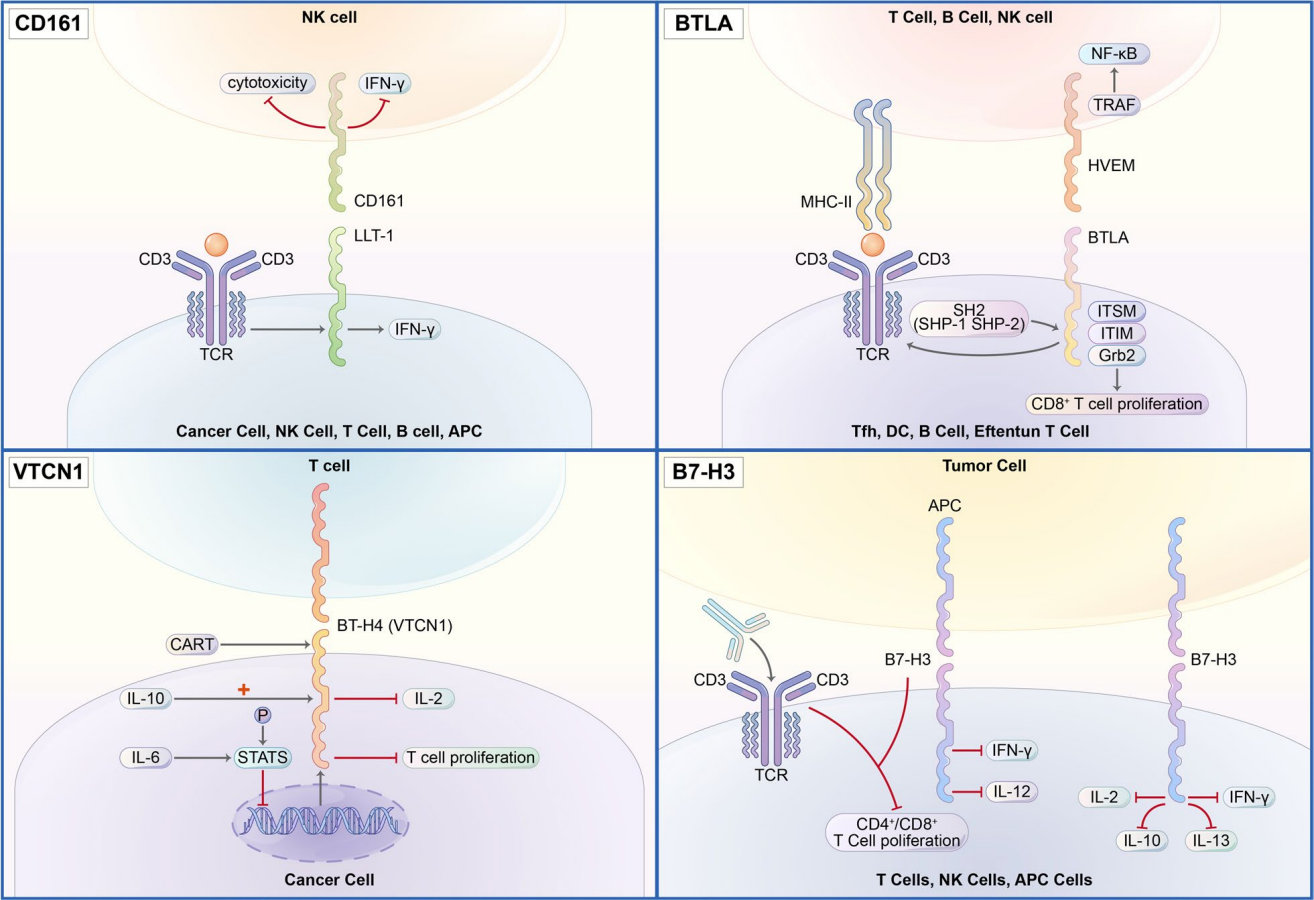
Mode of action of CD161, BTLA, VTCN1, and B7-H3 signaling pathways

CD161 (NKR-P1A) is widely found on NK cells, subsets of CD4+ and CD8+ T cells (Fig. 3). After binding to its ligand, LLT1 (lectin-like transcript 1), a C-type lectin-like receptor predominantly expressed on NK cells and T cell subsets, the CD161-LLT1 complex blocks the activation of NK cells [169]. Notably, LLT1 is expressed in immune cells. It enhances functions like targeting pathogens, presenting antigens to other cells, secreting cytokines, and improving the interactions between receptors and ligands on immune cells with co-stimulation. Another study showed that LLT1 expressed on NK cells induces the IFN-γ production, which further proved the vital role of LLT1 in pathogens-targeted early innate immune response [170].

## B and T lymphocyte attenuator (BTLA)

BTLA is a coinhibitory receptor that works with HVEM, which structurally belongs to the immunoglobulin (Ig) superfamily. It is widely expressed in T cells, B cells, and DCs [171]. The cytoplasmic domain of BTLA consists of three motifs: an ITIM, an ITSM, and a growth factor receptor-bound protein 2 motif (Grb2) [172]. Src homology 2 (SH2), which contains phosphatase 1 and 2 (SHP-1 and SHP-2), was recruited to ITIM and ITSM motifs through the ligation of BTLA by HVEM, resulting in the suppressed activity of TCR [171]. BTLA also contains a Grb2 binding site that promotes CD8+ T cell cytokine production and proliferation [173]. Thus, the signaling actions of BTLA should be defined on cell types since they may vary between different cell populations. There are limited clinical trials on BTLA. JS004, also named TAB004, is the only available recombinant humanized mAb specifically targeting BTLA developed by Shanghai Junshi Bioscience Co., Ltd. Some latest studies focused on BTLA’s effect in advanced solid tumors (NCT04278859, NCT04477772, NCT04929080).

## VTCN1

VTCN1 (B7-H4), also called B7x/B7s, is a B7 superfamily member identified as an inhibitory modulator of T cell response [174] through interacting with an unknown receptor. B7-H4 was first discovered on antigen-presenting cells [175]. The mRNA encoding B7-H4 is widely found in murine and human peripheral tissues. However, the B7-H4 cell surface protein is limited to normal human epithelial cells of the female genital tract, kidney [176], lung, and pancreas [177]. The regulation of B7-H4 in humans has two main mechanisms. In monocytes, macrophages, and myeloid DCs, IL-6 and IL-10 can promote the expression of B7-H4. However, the promotion can be interrupted with granulocyte–macrophage colony-stimulating factor (GM-CSF) and IL-4 [174].

### Clinical trials on B7-H4 (Table 5)

Since the receptor of B7-H4 remains unknown, only three studies are currently registered on ClinicalTrials.gov.

- An ongoing trial uses anti-B7-H4 antibody FPA150 as a single agent or cooperated with Pembrolizumab in patients with advanced solid tumors (NCT03514121). Twenty-nine patients were enrolled. Researchers did not report any DLTs or grade 4/5 toxicities, ORR was 3%, and DCR was 38% in combination therapy, making FPA150 a good choice for advanced solid cancer tumors.

## B7-H3

It is also named CD276 and has several isoforms. Due to different splicing, there are 4Ig domain transcripts (VCVC) and 2Ig domain transcripts (V1C2). Only the isoform of B7-H3 with a single VC domain is expressed in mice. The B7-H3 mRNA is widely expressed in lymphoid and non-lymphoid organs with relatively low protein levels. An abnormal B7-H3 rocketing is always associated with tumorigenesis and inflammation. Evidence supports the costimulatory and coinhibitory receptors for B7-H3 [57].

### Clinical trials on B7-H3 (Table 5)

Exciting news came from Enoblituzumab (MGA271), an engineered Fc humanized IgG1 mAb against B7-H3, where Enoblituzumab was used for participants with localized intermediate and high-risk prostate cancer before performing radical prostatectomy (NCT02923180). Thirty-two participants received treatment, among which most patients only experienced grade 1 (*n* = 31) or grade 2 (*n* = 12) treatment-related adverse events, and only four underwent grade 3 adverse events. By the way, we could see cardiac disorders as major serious adverse events in the study: 1 case with myocarditis and 1 with pericardial effusion). The result showed that 31% of patients had a more than 10% decline in PSA before post-prostatectomy, and an altered Gleason score was observed, indicating the efficacy of Enoblituzumab for prostate cancer patients.

- In another study (NCT02475213), researchers tried a combination therapy of Enoblituzumab plus Pembrolizumab on patients with advanced solid cancer, including melanoma, NSCLC, HNSCC, and urothelial cancer. One hundred and thirty-three participants joined the phase I study. They all received ≥ 1 dose of study treatment and failed to reach the maximum tolerated dose of Enoblituzumab with Pembrolizumab at 2 mg/kg. In the phase II study, 67 participants (including 21 with HNSCC, 16 with NSCLC, 17 with UC, and 13 with melanoma) were given intravenous Enoblituzumab (15 mg/kg) every three weeks plus Pembrolizumab (2 mg/kg) every three weeks. The result showed that 116 patients (87.2%) suffered treatment-related adverse events, and the percentage was 28.6% for grades ≥ 3. One treatment-related death occurred (pneumonitis). Objective responses occurred in 6 of 18 (33.3% [95% CI 13.3 to 59.0]) patients with checkpoint inhibitor (CPI)-naïve HNSCC and in 5 of 14 (35.7% [95% CI 12.8 to 64.9]) patients with CPI-naïve NSCLC [178]. This phase I/II trial showed that combining Enoblituzumab with Pembrolizumab demonstrated acceptable safety and effect in patients with CPI-naïve HNSCC and NSCLC.

An agent MGD009 (NCT02628535) is a humanized B7-H3/CD3 dual-affinity re-Targeting (DART) protein. For unknown reasons, the project has been terminated. The same team turned to MGD009 administered in combination with MGA012 (Anti-PD-1Antibody) (NCT03406949). This trial ended without an exact explanation.

## TIGIT

TIGIT belongs to the PVR‐like proteins family, first reported in 2008 through a genomic search for T-cell-specific genes that encode potential inhibitory receptors. It has an extracellular immunoglobulin (Ig) variable domain, a type 1 transmembrane domain, and a cytoplasmic tail with two inhibitory motifs: an ITIM and an Ig tail-tyrosine (ITT)-like motif. TIGIT has three ligands, CD155, CD112, and CD113, which all belong to a family of nectin and NECL molecules. Among the three ligands, TIGIT has the highest affinity with CD155. Once TIGIT is binding to CD155, the ITT-like motif would be phosphorylated, phosphatase-1 (SHIP-1) would be gathered, and finally, inhibiting IFN-γ production by NK cells. Besides, TIDIT could bind CD155 with higher affinity than CD226, the latter being the costimulatory receptor. All in all, TIGIT inhibits immunity through multiple mechanisms.

### Clinical trials on TIGIT (Table 5)

Several clinical trials on TIGIT are ongoing or completed on Clinicaltrials.gov, including Tiragolumab, Etigilimab (OMP-313M32), etc.

A phase II study recently released its result (NCT03563716, CITYSCAPE). Totally, 155 patients with NSCLC were enrolled. They were given Atezolizumab monotherapy (with placebo, 68 patients) or combination therapy of Tiragolumab plus Atezolizumab (67 patients). Objective response occurred in 21 patients (31.3% [95% CI 19.5–43.2]) in the Tiragolumab plus Atezolizumab group versus 11 patients (16.2% [6.7–25.7]) in the placebo plus Atezolizumab group (*p* = 0.031), and median PFS was 5.4 months and 3.6 months (HR = 0.57), respectively. Fourteen (21%) patients receiving combination therapy and 12 (18%) patients receiving monotherapy had serious adverse effects. In patients with high PD-L1 expression (TPS ≥50%), the improvement effect of Tiragolumab combined with Atezolizumab was more obvious, ORR: 55.2% vs 17.2%, HR = 0.33. The most frequently reported grade 3 or worse treatment-related adverse event was lipase increase (in six [9%] patients with combination therapy against two [3%] with monotherapy). Two treatment-related deaths (including pyrexia and infection) occurred in the combination therapy group [179]. This trial showed that Tiragolumab plus Atezolizumab improved ORR and PFS compared with Atezolizumab monotherapy in PD-L1 positive NSCLC.

Several phase III studies on Tiragolumab have been conducted (SKYSCRAPER-01, NCT04294810; SKYSCRAPER-02, NCT04256421; SKYSCRAPER-02C, NCT04665856), targeting NSCLC and small-cell lung carcinoma relatively. However, the results did not meet our expectations.

On May 11, Genentech announced that the study of SKYSCRAPER-01 did not meet its co-primary endpoint of progression-free survival. At this first analysis, the other co-primary endpoint of overall survival (OS) was immature, and the trial phase of TIGIT immunotherapy in combination with first-line PD-L1 non-small-cell therapy failed.

ASCO reported the result of the study NCT04256421. In this study, 490 eligible patients with untreated extensive-stage small-cell lung cancer (ES-SCLC) were randomized 1:1 to receive Atezolizumab, carboplatin, and etoposide with or without Tiragolumab (*n* = 243, *n* = 247). The result showed that the mPFS of the experimental group is 5.4m (95% CI 4.7–5.5), and that is 5.6m (95% CI 5.4–5.9) of the placebo group; mOS is 13.6m (95%CI 10.8–14.9) vs 13.6m (95%CI 12.3–15.2), and grade 3/4 TRAEs occurred in 52.3% of the experimental group and 55.7% of the placebo group and Grade 5 TRAEs occurred in 0.4% of experimental group and 2.0% placebo group [180]. The result showed that Tiragolumab combined with Atezolizumab plus chemotherapy (carboplatin/etoposide) did not improve the efficacy of ES-SCLC.

Despite the failure of Tiragolumab phase III studies, the search for TIGIT targets continues. In another phase I study (NCT03119428), 33 patients with locally advanced or metastatic cancer were enrolled (Phase Ia, *n* = 23. Phase Ib, *n* = 10). In phase Ia study, patients underwent 14-day treatment cycles with anti-TIGIT antibody Etigilimab alone (0.3, 1.0, 3.0, 10.0, 20.0 mg/kg IV) while in the phase Ib study, patients received a combination therapy of Nivolumab and Etigilimab (3.0, 10.0, 20.0 mg/kg Etigilimab and 240 mg Nivolumab). The study was terminated for a sponsor decision but still released part of the result. It reported no DLT detected. The maximum administered dose was 20 mg/kg. At the same time, MTD for monotherapy and combination therapy was not determined. The median PFS was 56.0 days and 57.5 days for phase Ia and phase Ib, respectively. Besides, in the phase Ia study, seven patients (30.0%) had SD, and no PR was reported; in the phase Ib study, one patient had a partial response. One patient had prolonged SD of nearly eight months. The most reported AEs were rash (43.5%), nausea (34.8%), and fatigue (30.4%) in phase Ia and decreased appetite (50.0%), nausea (50.0%), and rash (40%) in Phase Ib. Six patients experienced Grade ≥3 treatment-related AEs [181].

At least 50 clinical trials are registered on Clinicaltrials.gov, though only two studies have yielded results. Etigilimab has not reached phase III study, but recent outcomes indicated its potential for cancer therapy. NSCLC is a good target, and more combination therapies on Tiragolumab are appreciated.

## Conclusion

The success of PD-1/L1 and CTLA-4 blockade contributed to the research enthusiasm for finding additional agents and inhibitory pathways that could help improve malignancy management. However, despite the significant advancement of ICI therapy, several challenges, including the immune-associated adverse events, treatment duration, biomarkers to predict treatment efficacy, and most importantly, the treatment resistance and limited clinical benefit to a minority of patients, remain to be addressed.

ICI therapy is often related to toxicities caused mainly by increased immune system activity. These toxicities may present as systemic symptoms, including fatigue, hypothyroidism, anemia, neutropenia, rash, colitis, and pneumonitis. Some immune-associated adverse events (myocarditis and hepatitis) might even be fatal. Therefore, the successful application of ICI therapy demands a comprehensive understanding of these toxicities from clinicians and oncologists to promptly prevent, accurately recognize, and appropriately treat each adverse event. Most adverse events of level 1 could be resolved by expectant treatment. Most adverse events of levels two and three could be resolved by interrupting the treatment and administrating the short course of steroids. In contrast, some severe adverse events of level 4 might require the impact therapy of steroids and even the administration of Infliximab if hormonotherapy failed [182].

Besides, given the relatively favorable safety profile of ICI therapy, dose selection has been at the higher end of the dose range, well above the lowest possible effective dose, as well as the dosing interval and the duration of treatment. Establishing the labeled dose of ICI could potentially increase the treatment outcome. Some studies have speculated that interindividual pharmacokinetic differences in clearance rates influence the results and that individual dosing should be considered [183]. In addition, as ICIs function as reactivators of anti-tumor immunity, one question is whether there is the possibility of extending the dosing intervals once the immune response has been restored. Moreover, the current maintenance treatment of ICI therapy usually lasts 2 years, and whether more extended maintenance treatment increases efficacy remains unknown.

So far, the response rates to ICI therapy remain low. Most patients are observed with primary resistance. Some responders are observed with acquired resistance after the initial response. The treatment resistance could be attributed to tumor intrinsic and microenvironment extrinsic factors [184]. Tumor innate mechanisms are mainly categorized into the alteration of antigen-presenting machinery (loss of tumor antigen and MHC) and alteration of immunosuppressive pathways (PI3K, WNT/b-catenin, and IFN-γ). Extrinsic mechanisms mainly depend on the inhibitory effects of immune response from immunosuppressive cells in the microenvironment. An in-depth understanding of these mechanisms would help design new strategies and combination therapies to overcome resistance.

Therefore, developing novel immune checkpoint molecules with better treatment efficacy and the potential of combined therapy to overcome treatment resistance is necessary. Nowadays, novel inhibitory checkpoints are being widely investigated to expand the application and effectiveness of existing ICI therapy. Clinical trials on LAG-3 pile up due to its promising efficacy. More trials with CD38/CD39/CD73 are needed since they differ in the immune modulatory mechanisms. Once they’re applied in combination with other drugs, antagonism is less possible. However, there are still several puzzles remaining to be solved.

Firstly, reports on the combination of drug usage are far from satisfying. A fully described and systematic program isn’t available. As combined therapy could be used as the initial treatment and sequential therapy after the classical PD-1/PD-L1/CTLA-4 treatment resistance, more combination strategies are urgently needed. Anti-PD-1 combined with anti-TIM-3 or anti-BTLA deserves further exploring as evidence suggests that BTLA, a receptor structurally related to PD-1, could contribute to resistance to anti-PD-1 therapy [185]. Likewise, a co-blockade of TIM-3 and PD-1 may result in tumor regression in preclinical models and improve anti-tumor T cell responses in patients with advanced cancers [50]. Besides, a combination of anti-CD39 and anti-CD73 is worth further exploring as they both function as ectonucleotidases that interfere with anti-tumor immune responses.

Secondly, the ligands for some checkpoint molecules (e.g., CD161 and CD93) must be fully identified, which could help develop novel targeted drugs and significantly increase the therapeutic efficacy.

Thirdly, in the last two decades, we excitedly witnessed a long list of antibodies emerge and gradually move into clinical trials. However, till now, very few drugs have taken that step. More phase II and III trials are expected to identify clinical application value completely.

Fourthly, as a good form of immunotherapy, CAR-T therapy based on gene-editing technology has been rapidly developing in recent years, showing remarkable results in clinical applications and bringing a new dawn to personalized treatment for tumor patients. More trials are expected to test the feasibility of combining ICIs and CAR-T therapy.

Lastly, the clinical response to ICI therapy varies among patients, and heterogeneous responses have been observed in different tumor regions of the patient. Thus, several biomarkers have been identified and proposed to predict immunotherapy responses. Currently, PD-L1 expression, CD8+ T cell infiltration, tumor mutation burden (TMB), microsatellite instability (MSI), and IFN-γ have been the most widely used biomarkers [186]. Future development of effective predictive biomarkers would undeniably help select patients likely to benefit from ICI therapy. As ICI therapy has still been a massive cost in many developing countries, selecting beneficiaries would also deal with overtreatment.

## Data Availability

Not applicable.

## Abbreviations

ICI: Immune checkpoint inhibitor
PD-1: Programmed cell death protein 1
PD-L1: Programmed cell death ligand 1
CTLA-4: Cytotoxic T lymphocyte-associated antigen-4
VISTA: V-domain Ig suppressor of T cell activation
PD-1H: PD-1 homolog
CAR-T: Chimeric antigen receptor T
MHC: Major histocompatibility complex
APC: Antigen-presenting cell
TCR: T cell receptor
TME: Tumor microenvironment
TIM-3: T cell immunoglobulin and mucin domain-containing protein 3
LAG-3: Lymphocyte activation gene 3
IDO-1: Indoleamine 2,3-dioxygenase 1
TIL: Tumor-infiltrating lymphocyte
Th: Helper T
Treg: Regulatory T
NSCLC: Non-small-cell lung cancer
PFS: Progression-free survival
CLEC2D: C-type lectin domain family 2 member D
IFN-γ: Interferon-γ
ENTPD1: Ecto-nucleoside triphosphate diphosphohydrolase-1
ADPR: Adenosine diphosphate ribose
CDKN2B: Cyclin-dependent kinase inhibitor 2B
AML: Acute myeloid leukemia
DC: Dendritic cell
HIF: Hypoxia-induced factor
TGF: Transforming growth factor
EMT: Epithelial-to-mesenchymal transition
HMGB1: High-mobility group protein 1
NKTCL: Natural killer/T cell lymphomas
RCC: Renal cell cancer
VHL: Von Hippel–Lindau gene
ITIM: Immune receptor tyrosine-based inhibitory motif
ITSM: Immune receptor tyrosine-based switch motif
BAT3: HLA-B-associated transcript 3
SIRPA: Signal regulatory protein α
LLT1: Lectin-like transcript 1
BTLA: B and T lymphocyte attenuator

## References

[1] Xia L, Liu Y, Wang Y (2019). PD-1/PD-L1 blockade therapy in advanced non-small-cell lung cancer: current status and future directions. Oncologist.

[2] Darvin P, Toor SM, SasidharanNair V, Elkord E (2018). Immune checkpoint inhibitors: recent progress and potential biomarkers. Exp Mol Med.

[3] Huang PW, Chang JW (2019). Immune checkpoint inhibitors win the 2018 Nobel Prize. Biomed J.

[4] Topalian SL, Taube JM, Anders RA, Pardoll DM (2016). Mechanism-driven biomarkers to guide immune checkpoint blockade in cancer therapy. Nat Rev Cancer.

[5] Waldman AD, Fritz JM, Lenardo MJ (2020). A guide to cancer immunotherapy: from T cell basic science to clinical practice. Nat Rev Immunol.

[6] Rabinovich GA, Gabrilovich D, Sotomayor EM (2007). Immunosuppressive strategies that are mediated by tumor cells. Annu Rev Immunol.

[7] Scanlan MJ, Gure AO, Jungbluth AA, Old LJ, Chen YT (2002). Cancer/testis antigens: an expanding family of targets for cancer immunotherapy. Immunol Rev.

[8] Stone JD, Harris DT, Kranz DM (2015). TCR affinity for p/MHC formed by tumor antigens that are self-proteins: impact on efficacy and toxicity. Curr Opin Immunol.

[9] Jiang T (2019). Tumor neoantigens: from basic research to clinical applications. J Hematol Oncol.

[10] Ribas A, Wolchok JD (2018). Cancer immunotherapy using checkpoint blockade. Science.

[11] Hmeljak J (2018). Integrative molecular characterization of malignant pleural mesothelioma. Cancer Discov.

[12] Rowshanravan B, Halliday N, Sansom DM (2018). CTLA-4: a moving target in immunotherapy. Blood.

[13] Zhao Y (2019). PD-L1:CD80 cis-heterodimer triggers the co-stimulatory receptor CD28 while repressing the inhibitory PD-1 and CTLA-4 pathways. Immunity.

[14] Hodi FS (2010). Improved survival with Ipilimumab in patients with metastatic melanoma. N Engl J Med.

[15] Gao X, McDermott DF (2018). Ipilimumab in combination with Nivolumab for the treatment of renal cell carcinoma. Expert Opin Biol Ther.

[16] Du X (2018). A reappraisal of CTLA-4 checkpoint blockade in cancer immunotherapy. Cell Res.

[17] Jiang Y, Chen M, Nie H, Yuan Y (2019). PD-1 and PD-L1 in cancer immunotherapy: clinical implications and future considerations. Hum Vaccin Immunother.

[18] Sun C, Mezzadra R, Schumacher TN (2018). Regulation and function of the PD-L1 checkpoint. Immunity.

[19] Shi J (2018). PD-1 controls follicular T helper cell positioning and function. Immunity.

[20] Gordon SR (2017). PD-1 expression by tumour-associated macrophages inhibits phagocytosis and tumour immunity. Nature.

[21] Kwok G, Yau TC, Chiu JW, Tse E, Kwong YL (2016). Pembrolizumab (Keytruda). Hum Vaccin Immunother.

[22] Abdel-Wahab N, Shah M, Suarez-Almazor ME (2016). Adverse events associated with immune checkpoint blockade in patients with cancer: a systematic review of case reports. PLoS ONE.

[23] Barroso-Sousa R (2018). Incidence of endocrine dysfunction following the use of different immune checkpoint inhibitor regimens: a systematic review and meta-analysis. JAMA Oncol.

[24] Palmieri DJ, Carlino MS (2018). Immune checkpoint inhibitor toxicity. Curr Oncol Rep.

[25] Akturk HK (2019). Immune checkpoint inhibitor-induced Type 1 diabetes: a systematic review and meta-analysis. Diabet Med.

[26] Schoenfeld AJ, Hellmann MD (2020). Acquired resistance to immune checkpoint inhibitors. Cancer Cell.

[27] Zhang H (2021). Regulatory mechanisms of immune checkpoints PD-L1 and CTLA-4 in cancer. J Exp Clin Cancer Res.

[28] Zappasodi R, Merghoub T, Wolchok JD (2018). Emerging concepts for immune checkpoint blockade-based combination therapies. Cancer Cell.

[29] Wolchok JD (2013). Nivolumab plus Ipilimumab in advanced melanoma. N Engl J Med.

[30] Reck M (2019). Nivolumab plus Ipilimumab versus chemotherapy as first-line treatment in advanced non-small-cell lung cancer with high tumour mutational burden: patient-reported outcomes results from the randomised, open-label, phase III CheckMate 227 trial. Eur J Cancer.

[31] Baas P (2021). First-line Nivolumab plus Ipilimumab in unresectable malignant pleural mesothelioma (CheckMate 743): a multicentre, randomised, open-label, phase 3 trial. Lancet.

[32] Kaseb AO (2022). Perioperative Nivolumab monotherapy versus Nivolumab plus Ipilimumab in resectable hepatocellular carcinoma: a randomised, open-label, phase 2 trial. Lancet Gastroenterol Hepatol.

[33] Cohen R (2020). RECIST and iRECIST criteria for the evaluation of Nivolumab plus Ipilimumab in patients with microsatellite instability-high/mismatch repair-deficient metastatic colorectal cancer: the GERCOR NIPICOL phase II study. J Immunother Cancer.

[34] Cella D (2019). Patient-reported outcomes of patients with advanced renal cell carcinoma treated with Nivolumab plus Ipilimumab versus sunitinib (CheckMate 214): a randomised, phase 3 trial. Lancet Oncol.

[35] Larkin J (2015). Combined nivolumab and ipilimumab or monotherapy in untreated melanoma. N Engl J Med.

[36] Lim JY, Gerber SA, Murphy SP, Lord EM (2014). Type I interferons induced by radiation therapy mediate recruitment and effector function of CD8(+) T cells. Cancer Immunol Immunother.

[37] Twyman-Saint Victor C (2015). Radiation and dual checkpoint blockade activate non-redundant immune mechanisms in cancer. Nature.

[38] Deng L (2014). Irradiation and anti-PD-L1 treatment synergistically promote antitumor immunity in mice. J Clin Invest.

[39] Wang X (2017). Suppression of type I IFN signaling in tumors mediates resistance to Anti-PD-1 treatment that can be overcome by radiotherapy. Cancer Res.

[40] Formenti SC (2018). Radiotherapy induces responses of lung cancer to CTLA-4 blockade. Nat Med.

[41] Langer CJ (2016). Carboplatin and pemetrexed with or without Pembrolizumab for advanced, non-squamous non-small-cell lung cancer: a randomised, phase 2 cohort of the open-label KEYNOTE-021 study. Lancet Oncol.

[42] Horinouchi H (2021). Pembrolizumab plus pemetrexed-platinum for metastatic nonsquamous non-small-cell lung cancer: KEYNOTE-189 Japan Study. Cancer Sci.

[43] Reck M (2021). First-line nivolumab plus ipilimumab versus chemotherapy in advanced NSCLC With 1% or greater tumor PD-L1 expression: patient-reported outcomes from CheckMate 227 Part 1. J Thorac Oncol.

[44] Ariyan CE (2018). Robust antitumor responses result from local chemotherapy and CTLA-4 blockade. Cancer Immunol Res.

[45] ElTanbouly MA (2020). VISTA is a checkpoint regulator for naive T cell quiescence and peripheral tolerance. Science.

[46] Flies DB (2014). Coinhibitory receptor PD-1H preferentially suppresses CD4(+) T cell-mediated immunity. J Clin Invest.

[47] Wang L (2014). Disruption of the immune-checkpoint VISTA gene imparts a proinflammatory phenotype with predisposition to the development of autoimmunity. Proc Natl Acad Sci U S A.

[48] Flies DB, Higuchi T, Chen L (2015). Mechanistic assessment of PD-1H coinhibitory receptor-induced T cell tolerance to allogeneic antigens. J Immunol.

[49] Mathewson ND (2021). Inhibitory CD161 receptor identified in glioma-infiltrating T cells by single-cell analysis. Cell.

[50] Wolf Y, Anderson AC, Kuchroo VK (2020). TIM3 comes of age as an inhibitory receptor. Nat Rev Immunol.

[51] Wang J (2019). Fibrinogen-like protein 1 is a major immune inhibitory ligand of LAG-3. Cell.

[52] Horenstein AL, Faini AC, Malavasi F (2021). CD38 in the age of COVID-19: a medical perspective. Physiol Rev.

[53] Beldi G (2008). Regulated catalysis of extracellular nucleotides by vascular CD39/ENTPD1 is required for liver regeneration. Gastroenterology.

[54] Hershberger KA, Martin AS, Hirschey MD (2017). Role of NAD(+) and mitochondrial sirtuins in cardiac and renal diseases. Nat Rev Nephrol.

[55] Chen JF, Eltzschig HK, Fredholm BB (2013). Adenosine receptors as drug targets—what are the challenges?. Nat Rev Drug Discov.

[56] Du H (2019). Antitumor responses in the absence of toxicity in solid tumors by targeting B7–H3 via chimeric antigen receptor T cells. Cancer Cell.

[57] Schildberg FA, Klein SR, Freeman GJ, Sharpe AH (2016). Coinhibitory pathways in the B7-CD28 ligand-receptor family. Immunity.

[58] Iwasaki M, Liedtke M, Gentles AJ, Cleary ML (2015). CD93 marks a non-quiescent human leukemia stem cell population and is required for development of MLL-rearranged acute myeloid leukemia. Cell Stem Cell.

[59] Yuan L, Tatineni J, Mahoney KM, Freeman GJ (2021). VISTA: a mediator of quiescence and a promising target in cancer immunotherapy. Trends Immunol.

[60] ElTanbouly MA, Schaafsma E, Noelle RJ, Lines JL (2020). VISTA: Coming of age as a multi-lineage immune checkpoint. Clin Exp Immunol.

[61] Gao J (2017). VISTA is an inhibitory immune checkpoint that is increased after Ipilimumab therapy in patients with prostate cancer. Nat Med.

[62] Huang X (2020). VISTA: an immune regulatory protein checking tumor and immune cells in cancer immunotherapy. J Hematol Oncol.

[63] Mehta N (2020). An engineered antibody binds a distinct epitope and is a potent inhibitor of murine and human VISTA. Sci Rep.

[64] Sasikumar PG (2021). PD-1 derived CA-170 is an oral immune checkpoint inhibitor that exhibits preclinical anti-tumor efficacy. Commun Biol.

[65] Nowak EC (2017). Immunoregulatory functions of VISTA. Immunol Rev.

[66] Cronstein BN, Sitkovsky M (2017). Adenosine and adenosine receptors in the pathogenesis and treatment of rheumatic diseases. Nat Rev Rheumatol.

[67] Warburg O (1956). On respiratory impairment in cancer cells. Science.

[68] Boison D, Yegutkin GG (2019). Adenosine metabolism: emerging concepts for cancer therapy. Cancer Cell.

[69] Li L (2018). Metformin-induced reduction of CD39 and CD73 blocks myeloid-derived suppressor cell activity in patients with ovarian cancer. Cancer Res.

[70] Guo X (2018). Immunosuppressive effects of hypoxia-induced glioma exosomes through myeloid-derived suppressor cells via the miR-10a/Rora and miR-21/Pten Pathways. Oncogene.

[71] Allard D, Allard B, Stagg J (2020). On the mechanism of anti-CD39 immune checkpoint therapy. J Immunother Cancer.

[72] Yu M (2020). CD73 on cancer-associated fibroblasts enhanced by the A2B-mediated feedforward circuit enforces an immune checkpoint. Nat Commun.

[73] Li XY (2019). Targeting CD39 in cancer reveals an extracellular ATP- and inflammasome-driven tumor immunity. Cancer Discov.

[74] Green DR, Ferguson T, Zitvogel L, Kroemer G (2009). Immunogenic and tolerogenic cell death. Nat Rev Immunol.

[75] Chen Y (2006). ATP release guides neutrophil chemotaxis via P2Y2 and A3 receptors. Science.

[76] Zumerle S (2019). Intercellular calcium signaling induced by ATP potentiates macrophage phagocytosis. Cell Rep.

[77] Chen S (2019). CD73: an emerging checkpoint for cancer immunotherapy. Immunotherapy.

[78] Neo SY (2020). CD73 immune checkpoint defines regulatory NK cells within the tumor microenvironment. J Clin Invest.

[79] Wennerberg E (2020). CD73 blockade promotes dendritic cell infiltration of irradiated tumors and tumor rejection. Cancer Immunol Res.

[80] Chatterjee S (2018). CD38-NAD(+)Axis regulates immunotherapeutic anti-tumor T cell response. Cell Metab.

[81] Chmielewski JP (2018). CD38 inhibits prostate cancer metabolism and proliferation by reducing cellular NAD(+) pools. Mol Cancer Res.

[82] Mottahedeh J (2018). CD38 is methylated in prostate cancer and regulates extracellular NAD+. Cancer Metab.

[83] Malavasi F (2008). Evolution and function of the ADP ribosyl cyclase/CD38 gene family in physiology and pathology. Physiol Rev.

[84] Facon T (2021). Daratumumab, lenalidomide, and dexamethasone versus lenalidomide and dexamethasone alone in newly diagnosed multiple myeloma (MAIA): overall survival results from a randomised, open-label, phase 3 trial. Lancet Oncol.

[85] Mateos MV (2020). Overall survival with Daratumumab, bortezomib, Melphalan, and Prednisone in newly diagnosed multiple myeloma (ALCYONE): a randomised, open-label, phase 3 trial. Lancet.

[86] Moreau P (2019). Bortezomib, thalidomide, and dexamethasone with or without Daratumumab before and after autologous stem-cell transplantation for newly diagnosed multiple myeloma (CASSIOPEIA): a randomised, open-label, phase 3 study. Lancet.

[87] Attal M (2019). Isatuximab plus pomalidomide and low-dose dexamethasone versus pomalidomide and low-dose dexamethasone in patients with relapsed and refractory multiple myeloma (ICARIA-MM): a randomised, multicentre, open-label, phase 3 study. Lancet.

[88] Moreau P (2021). Isatuximab, carfilzomib, and dexamethasone in relapsed multiple myeloma (IKEMA): a multicentre, open-label, randomised phase 3 trial. Lancet.

[89] Huang H (2021). Daratumumab monotherapy for patients with relapsed or refractory natural killer/T-cell lymphoma, nasal type: an open-label, single-arm, multicenter, phase 2 study. J Hematol Oncol.

[90] Salles G (2019). Phase 2 study of daratumumab in relapsed/refractory mantle-cell lymphoma, diffuse large B-cell lymphoma, and follicular lymphoma. Clin Lymphoma Myeloma Leuk.

[91] Wang A (2021). Evaluation of preclinical activity of isatuximab in patients with acute lymphoblastic leukemia. Mol Cancer Ther.

[92] Spatola BN (2020). Fully human anti-CD39 antibody potently inhibits ATPase activity in cancer cells via uncompetitive allosteric mechanism. MAbs.

[93] Hargadon KM (2020). Tumor microenvironmental influences on dendritic cell and T cell function: A focus on clinically relevant immunologic and metabolic checkpoints. Clin Transl Med.

[94] Perrot I (2019). Blocking antibodies targeting the CD39/CD73 immunosuppressive pathway unleash immune responses in combination cancer therapies. Cell Rep.

[95] Zhai W (2020). A novel cyclic peptide targeting LAG-3 for cancer immunotherapy by activating antigen-specific CD8(+) T cell responses. Acta Pharm Sin B.

[96] Crise B, Rose JK (1992). Identification of palmitoylation sites on CD4, the human immunodeficiency virus receptor. J Biol Chem.

[97] Turner JM (1990). Interaction of the unique N-terminal region of tyrosine kinase p56lck with cytoplasmic domains of CD4 and CD8 is mediated by cysteine motifs. Cell.

[98] Topalian SL, Drake CG, Pardoll DM (2015). Immune checkpoint blockade: a common denominator approach to cancer therapy. Cancer Cell.

[99] Mastrangeli R, Micangeli E, Donini S (1996). Cloning of murine LAG-3 by magnetic bead bound homologous probes and PCR (gene-capture PCR). Anal Biochem.

[100] Iouzalen N, Andreae S, Hannier S, Triebel F (2001). LAP, a lymphocyte activation gene-3 (LAG-3)-associated protein that binds to a repeated EP motif in the intracellular region of LAG-3, may participate in the down-regulation of the CD3/TCR activation pathway. Eur J Immunol.

[101] Anderson AC, Joller N, Kuchroo VK (2016). Lag-3, Tim-3, and TIGIT: co-inhibitory receptors with specialized functions in immune regulation. Immunity.

[102] Schnell A, Bod L, Madi A, Kuchroo VK (2020). The yin and yang of co-inhibitory receptors: toward anti-tumor immunity without autoimmunity. Cell Res.

[103] Chocarro L (2021). Understanding LAG-3 signaling. Int J Mol Sci.

[104] Huard B, Prigent P, Pages F, Bruniquel D, Triebel F (1996). T cell major histocompatibility complex class II molecules down-regulate CD4+ T cell clone responses following LAG-3 binding. Eur J Immunol.

[105] Huard B, Tournier M, Hercend T, Triebel F, Faure F (1994). Lymphocyte-activation gene 3/major histocompatibility complex class II interaction modulates the antigenic response of CD4+ T lymphocytes. Eur J Immunol.

[106] Andrews LP, Marciscano AE, Drake CG, Vignali DA (2017). LAG3 (CD223) as a cancer immunotherapy target. Immunol Rev.

[107] Brignone C, Escudier B, Grygar C, Marcu M, Triebel F (2009). A phase I pharmacokinetic and biological correlative study of IMP321, a novel MHC class II agonist, in patients with advanced renal cell carcinoma. Clin Cancer Res.

[108] Tawbi HA (2022). Relatlimab and nivolumab versus nivolumab in untreated advanced melanoma. N Engl J Med.

[109] Mondanelli G (2017). A relay pathway between arginine and tryptophan metabolism confers immunosuppressive properties on dendritic cells. Immunity.

[110] Triplett TA (2018). Reversal of indoleamine 2,3-dioxygenase-mediated cancer immune suppression by systemic kynurenine depletion with a therapeutic enzyme. Nat Biotechnol.

[111] El-Zaatari M (2018). Indoleamine 2,3-dioxygenase 1, increased in human gastric pre-neoplasia, promotes inflammation and metaplasia in mice and is associated with type II hypersensitivity/autoimmunity. Gastroenterology.

[112] Giovannoni F (2020). AHR is a Zika virus host factor and a candidate target for antiviral therapy. Nat Neurosci.

[113] Minhas PS (2019). Macrophage de novo NAD(+) synthesis specifies immune function in aging and inflammation. Nat Immunol.

[114] Mitchell TC (2018). Epacadostat plus pembrolizumab in patients with advanced solid tumors: phase I results from a multicenter, open-label phase I/II trial (ECHO-202/KEYNOTE-037). J Clin Oncol.

[115] Turaj AH (2017). Antibody tumor targeting is enhanced by CD27 agonists through myeloid recruitment. Cancer Cell.

[116] Ghosh S (2020). Extended clinical and immunological phenotype and transplant outcome in CD27 and CD70 deficiency. Blood.

[117] Wang X, Dong C (2013). The CD70-CD27 axis, a new brake in the T helper 17 cell response. Immunity.

[118] Riether C (2017). CD70/CD27 signaling promotes blast stemness and is a viable therapeutic target in acute myeloid leukemia. J Exp Med.

[119] Shaffer DR (2011). T cells redirected against CD70 for the immunotherapy of CD70-positive malignancies. Blood.

[120] Remedios KA (2019). CD27 Promotes CD4(+) Effector T cell survival in response to tissue self-antigen. J Immunol.

[121] Croft M, Siegel RM (2017). Beyond TNF: TNF superfamily cytokines as targets for the treatment of rheumatic diseases. Nat Rev Rheumatol.

[122] Shaw J, Wang YH, Ito T, Arima K, Liu YJ (2010). Plasmacytoid dendritic cells regulate B-cell growth and differentiation via CD70. Blood.

[123] Ansell SM (2020). Safety and activity of Varlilumab, a novel and first-in-class agonist anti-CD27 antibody, for hematologic malignancies. Blood Adv.

[124] Burris HA (2017). Safety and activity of varlilumab, a novel and first-in-class agonist anti-CD27 antibody, in patients with advanced solid tumors. J Clin Oncol.

[125] Lim SH (2018). RIVA—a phase IIa study of Rituximab and Varlilumab in relapsed or refractory B-cell malignancies: study protocol for a randomized controlled trial. Trials.

[126] Nolte MA, van Olffen RW, van Gisbergen KP, van Lier RA (2009). Timing and tuning of CD27-CD70 interactions: the impact of signal strength in setting the balance between adaptive responses and immunopathology. Immunol Rev.

[127] Fehniger TA (2017). CD70 turns on NK cells to attack lymphoma. Blood.

[128] Croft M (2012). TNF superfamily in inflammatory disease: translating basic insights. Trends Immunol.

[129] Sawalha AH, Jeffries M (2007). Defective DNA methylation and CD70 overexpression in CD4+ T cells in MRL/lpr lupus-prone mice. Eur J Immunol.

[130] Zamora MR (2011). DNA viruses (CMV, EBV, and the herpesviruses). Semin Respir Crit Care Med.

[131] Karki R, Kanneganti TD (2019). Diverging inflammasome signals in tumorigenesis and potential targeting. Nat Rev Cancer.

[132] Jacobs J (2015). CD70: An emerging target in cancer immunotherapy. Pharmacol Ther.

[133] McEarchern JA (2008). Preclinical characterization of SGN-70, a humanized antibody directed against CD70. Clin Cancer Res.

[134] Diegmann J (2005). Identification of CD70 as a diagnostic biomarker for clear cell renal cell carcinoma by gene expression profiling, real-time RT-PCR and immunohistochemistry. Eur J Cancer.

[135] Ruf M (2015). pVHL/HIF-regulated CD70 expression is associated with infiltration of CD27+ lymphocytes and increased serum levels of soluble CD27 in clear cell renal cell carcinoma. Clin Cancer Res.

[136] Pal SK (2019). A phase 1 trial of SGN-CD70A in patients with CD70-positive, metastatic renal cell carcinoma. Cancer.

[137] De Meulenaere A (2021). An open-label, nonrandomized, phase Ib feasibility study of cusatuzumab in patients with nasopharyngeal carcinoma. Clin Transl Sci.

[138] Sadelain M, Brentjens R, Riviere I (2013). The basic principles of chimeric antigen receptor design. Cancer Discov.

[139] Wang QJ (2017). Preclinical evaluation of chimeric antigen receptors targeting CD70-expressing cancers. Clin Cancer Res.

[140] Park YP (2018). CD70 as a target for chimeric antigen receptor T cells in head and neck squamous cell carcinoma. Oral Oncol.

[141] de Mingo Pulido A (2018). TIM-3 regulates CD103(+) dendritic cell function and response to chemotherapy in breast cancer. Cancer Cell.

[142] Chihara N (2018). Induction and transcriptional regulation of the co-inhibitory gene module in T cells. Nature.

[143] Cao E (2007). T cell immunoglobulin mucin-3 crystal structure reveals a galectin-9-independent ligand-binding surface. Immunity.

[144] Campbell IG, Freemont PS, Foulkes W, Trowsdale J (1992). An ovarian tumor marker with homology to vaccinia virus contains an IgV-like region and multiple transmembrane domains. Cancer Res.

[145] Mushegian A (2002). Refining structural and functional predictions for secretasome components by comparative sequence analysis. Proteins.

[146] Oldenborg PA (2013). CD47: a cell surface glycoprotein which regulates multiple functions of hematopoietic cells in health and disease. ISRN Hematol.

[147] Oldenborg PA (2000). Role of CD47 as a marker of self on red blood cells. Science.

[148] Yi T (2015). Splenic dendritic cells survey red blood cells for missing Self-CD47 to trigger adaptive immune responses. Immunity.

[149] Lehrman EK (2018). CD47 protects synapses from excess microglia-mediated pruning during development. Neuron.

[150] Majeti R (2009). CD47 is an adverse prognostic factor and therapeutic antibody target on human acute myeloid leukemia stem cells. Cell.

[151] Oldenborg PA, Gresham HD, Lindberg FP (2001). CD47-signal regulatory protein alpha (SIRPalpha) regulates Fcgamma and complement receptor-mediated phagocytosis. J Exp Med.

[152] Gholamin S (2017). Disrupting the CD47-SIRPalpha anti-phagocytic axis by a humanized anti-CD47 antibody is an efficacious treatment for malignant pediatric brain tumors. Sci Transl Med.

[153] Chao MP (2010). Anti-CD47 antibody synergizes with Rituximab to promote phagocytosis and eradicate non-Hodgkin lymphoma. Cell.

[154] Okazawa H (2005). Negative regulation of phagocytosis in macrophages by the CD47-SHPS-1 system. J Immunol.

[155] Liu X (2015). CD47 blockade triggers T cell-mediated destruction of immunogenic tumors. Nat Med.

[156] Fujioka Y (1996). A novel membrane glycoprotein, SHPS-1, that binds the SH2-domain-containing protein tyrosine phosphatase SHP-2 in response to mitogens and cell adhesion. Mol Cell Biol.

[157] Kharitonenkov A (1997). A family of proteins that inhibit signalling through tyrosine kinase receptors. Nature.

[158] Xu MM (2017). Dendritic cells but not macrophages sense tumor mitochondrial DNA for cross-priming through signal regulatory protein alpha signaling. Immunity.

[159] Tsai RK, Discher DE (2008). Inhibition of "self" engulfment through deactivation of myosin-II at the phagocytic synapse between human cells. J Cell Biol.

[160] Veillette A, Thibaudeau E, Latour S (1998). High expression of inhibitory receptor SHPS-1 and its association with protein-tyrosine phosphatase SHP-1 in macrophages. J Biol Chem.

[161] Orr AW (2003). Low density lipoprotein receptor-related protein is a calreticulin coreceptor that signals focal adhesion disassembly. J Cell Biol.

[162] Chao MP (2010). Calreticulin is the dominant pro-phagocytic signal on multiple human cancers and is counterbalanced by CD47. Sci Transl Med.

[163] Woo SR (2014). STING-dependent cytosolic DNA sensing mediates innate immune recognition of immunogenic tumors. Immunity.

[164] Sikic BI (2019). First-in-human, first-in-class phase I trial of the anti-CD47 antibody Hu5F9-G4 in patients with advanced cancers. J Clin Oncol.

[165] Advani R (2018). CD47 Blockade by Hu5F9-G4 and rituximab in Non-Hodgkin's lymphoma. N Engl J Med.

[166] Lugano R (2018). CD93 promotes beta1 integrin activation and fibronectin fibrillogenesis during tumor angiogenesis. J Clin Invest.

[167] Barbera S (2019). The small GTPase Rab5c is a key regulator of trafficking of the CD93/Multimerin-2/beta1 integrin complex in endothelial cell adhesion and migration. Cell Commun Signal.

[168] Sun Y (2021). Blockade of the CD93 pathway normalizes tumor vasculature to facilitate drug delivery and immunotherapy. Sci Transl Med.

[169] Marrufo AM (2018). Blocking LLT1 (CLEC2D, OCIL)-NKRP1A (CD161) interaction enhances natural killer cell-mediated lysis of triple-negative breast cancer cells. Am J Cancer Res.

[170] Braud VM (2018). Expression of LLT1 and its receptor CD161 in lung cancer is associated with better clinical outcome. Oncoimmunology.

[171] Sakoda Y (2011). Dichotomous regulation of GVHD through bidirectional functions of the BTLA-HVEM pathway. Blood.

[172] Ritthipichai K (2017). Multifaceted role of BTLA in the control of CD8(+) T-cell fate after antigen encounter. Clin Cancer Res.

[173] Mintz MA (2019). The HVEM-BTLA axis restrains T cell help to germinal center B cells and functions as a cell-extrinsic suppressor in lymphomagenesis. Immunity.

[174] Dangaj D (2013). Novel recombinant human b7–h4 antibodies overcome tumoral immune escape to potentiate T-cell antitumor responses. Cancer Res.

[175] Xia F (2017). B7–H4 enhances the differentiation of murine leukemia-initiating cells via the PTEN/AKT/RCOR2/RUNX1 pathways. Leukemia.

[176] Chinnadurai R, Grakoui A (2010). B7–H4 mediates inhibition of T cell responses by activated murine hepatic stellate cells. Hepatology.

[177] Yi KH, Chen L (2009). Fine tuning the immune response through B7–H3 and B7–H4. Immunol Rev.

[178] Aggarwal C (2022). Dual checkpoint targeting of B7–H3 and PD-1 with Enoblituzumab and Pembrolizumab in advanced solid tumors: interim results from a multicenter phase I/II trial. J Immunother Cancer.

[179] Cho BC (2022). Tiragolumab plus Atezolizumab versus placebo plus Atezolizumab as a first-line treatment for PD-L1-selected non-small-cell lung cancer (CITYSCAPE): primary and follow-up analyses of a randomised, double-blind, phase 2 study. Lancet Oncol.

[180] Rudin CM (2022). SKYSCRAPER-02: Primary results of a phase III, randomized, double-blind, placebo-controlled study of Atezolizumab (atezo) + carboplatin + etoposide (CE) with or without Tiragolumab (tira) in patients (pts) with untreated extensive-stage small cell lung cancer (ES-SCLC). J Clin Oncol.

[181] Mettu NB (2022). A phase 1a/b open-label, dose-escalation study of etigilimab alone or in combination with nivolumab in patients with locally advanced or metastatic solid tumors. Clin Cancer Res.

[182] Champiat S (2016). Management of immune checkpoint blockade dysimmune toxicities: a collaborative position paper. Ann Oncol.

[183] Chatelut E, Le Louedec F, Milano G (2020). Setting the dose of checkpoint inhibitors: the role of clinical pharmacology. Clin Pharmacokinet.

[184] Sharma P, Hu-Lieskovan S, Wargo JA, Ribas A (2017). Primary, adaptive, and acquired resistance to cancer immunotherapy. Cell.

[185] Xu X (2020). PD-1 and BTLA regulate T cell signaling differentially and only partially through SHP1 and SHP2. J Cell Biol.

[186] Gibney GT, Weiner LM, Atkins MB (2016). Predictive biomarkers for checkpoint inhibitor-based immunotherapy. Lancet Oncol.

